# Addressing clinician moral distress: Implications from a mixed methods evaluation during Covid-19

**DOI:** 10.1371/journal.pone.0291542

**Published:** 2023-09-15

**Authors:** Jennifer A. Palmer, Megan Mccullough, Jolie Wormwood, Renda Soylemez Wiener, Nathan Mesfin, Michael Still, Chris S. Xu, Amy M. Linsky

**Affiliations:** 1 Center for Healthcare Organization and Implementation Research (CHOIR), VA Boston Healthcare System, Boston, Massachusetts, United States of America; 2 Department of Medicine, Boston University Chobanian & Avedisian School of Medicine, Boston, Massachusetts, United States of America; 3 Center for Healthcare Organization and Implementation Research (CHOIR), VA Bedford Medical Center, Bedford, Massachusetts, United States of America; 4 University of Massachusetts, Lowell, Massachusetts, United States of America; 5 University of New Hampshire, Durham, New Hampshire, United States of America; 6 Department of Medicine, VA Boston Healthcare System, Boston, Massachusetts, United States of America; 7 Department of Medicine, University of Minnesota Medical School, Minneapolis, Minnesota, United States of America; Houston Methodist Academic Institute, UNITED STATES

## Abstract

Clinician moral distress has been documented over the past several decades as occurring within numerous healthcare disciplines, often in relation to clinicians’ involvement in patients’ end-of-life decision-making. The resulting harms impact clinician well-being, patient well-being, and healthcare system functioning. Given Covid-19’s catastrophic death toll and associated demands on end-of-life decision-making processes, the pandemic represents a particularly important context within which to understand clinician moral distress. Thus, we conducted a convergent mixed methods study to examine its prevalence, associations with clinicians’ demographic and professional characteristics, and contributing circumstances among Veterans Health Administration (VA) clinicians. The study, conducted in April 2021, consisted of a cross-sectional on-line survey of VA clinicians at 20 VA Medical Centers with professional jurisdiction to place life-sustaining treatment orders working who were from a number of select specialties. The survey collected quantitative data on respondents’ demographics, clinical practice characteristics, attitudes and behaviors related to goals of care conversations, intensity of moral distress during “peak-Covid,” and qualitative data via an open-ended item asking for respondents to describe contributing circumstances if they had indicated any moral distress. To understand factors associated with heightened moral distress, we analyzed quantitative data using bivariate and multivariable regression analyses and qualitative data using a hybrid deductive/inductive thematic approach. Mixed methods analysis followed, whereby we compared the quantitative and qualitative datasets and integrated findings at the analytic level. Out of 3,396 eligible VA clinicians, 323 responded to the survey (9.5% adjusted response rate). Most respondents (81%) reported at least some moral distress during peak-Covid. In a multivariable logistic regression, female gender (OR 3.35; 95% CI 1.53–7.37) was associated with greater odds of moral distress, and practicing in geriatrics/palliative care (OR 0.40; 95% CI 0.18–0.87) and internal medicine/family medicine/primary care (OR 0.46; 95% CI 0.22–0.98) were associated with reduced odds of moral distress compared to medical subspecialties. From the 191 respondents who completed the open-ended item, five qualitative themes emerged as moral distress contributors: 1) patient visitation restrictions, 2) anticipatory actions, 3) clinical uncertainty related to Covid, 4) resource shortages, and 5) personal risk of contracting Covid. Mixed methods analysis found that quantitative results were consistent with these last two qualitative themes. In sum, clinician moral distress was prevalent early in the pandemic. This moral distress was associated with individual-, system-, and situation-level contributors. These identified contributors represent leverage points for future intervention to mitigate clinician moral distress and its negative outcomes during future healthcare crises and even during everyday clinical care.

## Introduction

Moral distress within healthcare occurs when internal or external conditions cause clinicians to provide care in ways that they feel have transgressed their ethical beliefs. Internal conditions may include fear of repercussions and self-doubt, and external conditions may include lack of support or hierarchies within the healthcare system [1]. Moral distress is sometimes used interchangeably with “moral injury”. The two constructs do have similarities; both relate to clinicians’ potential experience of having their moral integrity transgressed and their experience of associated negative psychological sequelae [2]. However, moral distress is a distinct construct; it is a precursor to the more severe experience of moral injury [3, 4] and may be more easily mitigated than moral injury when the causative internal and external conditions are addressed [2].

Moral distress within healthcare was initially described as a phenomenon several decades ago. Initial studies verified its impact within the field of nursing, with subsequent studies demonstrating similar effects across a wide variety of clinicians (e.g., physicians, psychologists, social workers, pharmacists, respiratory therapists) [5]. Impacts across disciplines include negative effects on clinician well-being, clinical practice, and healthcare system functioning. Clinicians may experience harm to their physical well-being (e.g., appetite loss, gastrointestinal symptoms, migraines) [6] and emotional well-being (e.g., anger, sadness, guilt, shame) [4, 7–11]. Clinician harms can translate into impaired clinical practice and endanger the quality and quantity of patient care, with potential for worsened patient health outcomes [5, 8, 11, 12]. Notably, clinician moral distress is associated with clinician burnout and turnover, which can negatively impact healthcare system functioning [8, 13, 14].

Given the magnitude of multi-level effects that can result from clinician moral distress, better understanding of contributing factors would enable development of mitigating interventions. Prior to the onset of the Covid-19 pandemic, the most frequently described source of moral distress across multiple clinical roles was related to end-of-life decision-making (e.g., goals of care, preferences for life-sustaining treatments) [8]. Specifically, moral distress arose when clinicians had to actively provide aggressive care that they felt was futile or unjustifiable [5, 13, 15]. Also pre-pandemic, nurses in surgical intensive care units reported higher levels of moral distress when patients lacked clear goals of care [15] and when the nurses were not integrated into patients’ end-of-life decision-making processes [13]. The pandemic, with its catastrophic toll of 1,134,710 deaths in the U.S. [16] and 6,950,655 deaths worldwide [17], and associated urgency for end-of-life decision-making, threatened to amplify levels of clinician moral distress.

We explored Covid-19-related moral distress among clinicians involved with end-of-life decision-making in the Veterans Health Administration (VA), the largest nationally integrated healthcare system in the U.S. We surveyed VA clinicians who were authorized to place life-sustaining treatment (LST) orders in the electronic health record. The parent study examined shared decision-making during goals of care conversations (GoCCs) in early phases of the pandemic; the survey also contained items about clinician moral distress. Thus, for this study, we conducted a convergent mixed methods analysis of heightened moral distress among VA clinicians early in the pandemic to understand its prevalence, association with clinicians’ demographic and professional characteristics, and emergent themes depicting the circumstances contributing to its manifestation.

## Methods

### Study setting

We administered a cross-sectional survey to healthcare clinicians from 20 VA Medical Centers. The centers were evenly distributed across the nation’s four census regions (of note: we intentionally stratified across geographic regions) and were selected for having experienced the greatest number of cumulative Covid-19 cases between March 2020 and October 2020.

This study was deemed exempt from VA Boston Healthcare System’s Institutional Review Board oversight, which instead was provided by The VA Boston Healthcare System’s Research and Development Committee. The study was granted a waiver of need to obtain written or oral informed consent from survey respondents.

### Study respondents and survey administration

We identified eligible clinicians from the VA Corporate Data Warehouse based on three criteria: 1) working as licensed independent clinicians (e.g., physicians, nurse practitioners, physician assistants) with the authority to place life-sustaining treatment (LST) orders in the electronic health record; 2) providing direct patient care in inpatient, outpatient, or long-term care settings; and 3) practicing in the discipline of internal medicine or its relevant subspecialties (e.g., cardiology, geriatrics, palliative care medicine, pulmonary and critical care), emergency medicine, family medicine, neurology, or surgery.

Between March and April 2021, we invited 3,396 potential respondents to complete the 33-item, electronic survey. Invitations were sent to institutional email addresses a maximum of three times, with the reminders sent to non-responders at weekly intervals. Participation was voluntary, and no incentive was offered. Authors did not have access to information that could identify individual respondents during or after data collection.

### Survey instrument

As detailed in S1 Table, we developed a survey by adapting two pre-existing instruments which focused on physicians’ attitudes and behaviors regarding GoCCs [18, 19]. The Binder et al. survey [18] was geared towards residents having code status discussions with patients outside of the ICU; the Brush et al. [19] survey intended to assess self-reported practices and attitudes amongst US critical care physicians regarding giving recommendations about limiting life support therapies. We extracted questions that addressed important elements of GoCCs (i.e., identifying healthcare proxy, discussing necessary medical information, discussing patients’ goals and values, and providing a recommendation) that could be applied broadly across many specialties who may be having these conversations with seriously ill patients during the Covid-19 pandemic. The intent was to understand how core elements of GoCCs might have changed during the Covid-19 pandemic, focusing on providers propensity for giving a recommendation. Given the novelty of the disease and prognostication uncertainty, we postulated that provision of recommendation might have been most impacted during the early portion of the pandemic.

All the questions were kept in the original format with the exception of minor wording changes. From the Binder survey, we used three questions: (1) identifying patients’ health-care proxy, (2) discussing the reversibility of code status decisions, and (3) providers’ self-confidence in delivering all the necessary medical information. We changed the “code status discussion” phrase to “goals of care conversation.” From the Brush survey, we used 36 questions that asked about physicians’ attitudes, behavior and specific approaches to GoCCs and recommendation provision. We altered the questions so that they referred to “providers” instead of “critical care physicians” and “patients” instead of “surrogate decision-makers.” Importantly, none of the questions were altered in a way that changed the substance of the question. Our final survey instrument is provided in S1 File.

#### Primary outcome: Moral distress

The survey included two items related to experiences of moral distress, which we defined as: “when professionals are unable to carry out what they believe to be ethically appropriate actions because of internal (e.g., fear of repercussions; self-doubt) or external (e.g., lack of support, hierarchies of healthcare system) constraints” [1]. First, a closed-ended item asked the respondent to rate the intensity of overall level of moral distress during peak-Covid, with response options spanning a 5-point Likert scale (i.e., “None”, “Mild”, “Uncomfortable”, “Intense”, “Severe”). We adapted this item from the VA Moral Distress Assessment Tool [20], a tool based upon two previously validated measures of moral distress [21, 22]. Any respondent who selected a response option other than “None” was presented with an optional open-ended item: “Can you tell us more about the circumstances that may have contributed to these feelings?”

#### General attitudes about goals of care conversations during peak-Covid-19

Respondents were instructed to identify a period of “peak-Covid-19,” defined as the period when workload, work hours, clinical demand, and Covid-19 cases were highest. Clinicians were then asked a series of questions about their attitudes related to GoCCs during peak-Covid-19. The survey had two items regarding *general attitudes about LST recommendations* during GoCCs, including perceived appropriateness (using a 4-point scale from “Very appropriate” to “Very inappropriate”) and comfort with giving LST recommendations (using a 4-point scale from “Very comfortable” to “Very uncomfortable”). Next, there were six items about *the general ethics of providing specific LST recommendations* (e.g., it unduly influences patients, it is only appropriate if wanted); response options were presented on a 4-point scale from “Disagree strongly” to “Agree strongly.” There were then nine items about the *ethical appropriateness of using specific dialogue techniques* during GoCCs (e.g., use vivid imagery, discuss small chance of recovery); response options were presented on a 4-point scale from “Definitely not appropriate” to “Definitely appropriate.”

*Specific attitudes and behaviors in goals of care conversations during peak-Covid*. The survey contained 12 items concerning Covid-specific GoCC attitudes and behaviors. Clinicians reported how often they asked patients with Covid if they wanted a recommendation about LST (e.g., cardiopulmonary resuscitation) decisions (5-point scale ranging from “Never” to “Always”). They also rated the perceived appropriateness of limiting a patient’s LST options (on a 4-point scale from “Definitely not appropriate” to “Definitely appropriate” because of the risk it poses to healthcare providers and because of limited resources for other patients, as well as how concerned they were (on a 5-point scale from “Not at all” to “Extremely”) about resource availability at their facility. Other survey items assessed comfort with prognosticating about whether patients will have outcomes consistent with their goals and values after respiratory failure for both patients with and without Covid (on a 4-point scale from “Very comfortable” to “Very uncomfortable”). Finally, a series of items addressed quality of GoCCs during peak-Covid, including how frequently clinicians felt confident that they provided patients with adequate information to make a fully informed decision (on a 5-point scale from “Never” to “Always”), whether they perceived the quality of their GoCCs to be better or worse compared to pre-pandemic, and the perceived impact of two specific factors on GoCC quality (i.e., restrictions on family/support presence and communicating over telephone). They also reported whether they had at least one GoCC during peak-Covid.

*Demographic and clinical practice characteristics*. The survey assessed several demographic (i.e., age, gender, race, ethnicity) and clinical practice characteristics [clinical role (i.e., fellow, resident, nurse practitioner or advanced practice nurse, and physician assistant), specialty (i.e., anesthesia, internal medicine, neurology, surgery, pulmonary medicine / critical care, cardiology, geriatrics, palliative care, emergency medicine, and other), year of graduation, percent clinical effort, and percent of clinical effort in inpatient and outpatient settings].

## Analysis

### Quantitative

We calculated descriptive statistics (counts and proportions) for all demographic and clinical practice characteristics, both for the entire sample (n = 323) and for the subsample (n = 191) who replied to the open-ended “moral distress” question. We also assessed the frequency and intensity of moral distress. For subsequent analyses, we dichotomized responses to the closed-ended moral distress question into “None”/”Mild”/”Uncomfortable” and “Intense”/”Severe,” with the latter representing heightened moral distress. Such dichotomization was driven by our research question’s focus on “heightened” levels of the moral distress outcome.

We examined associations between demographic and clinical practice characteristics with heightened moral distress during peak Covid-19. We used Chi-square or Kruskal-Wallis tests, as appropriate, to assess zero-order associations of each demographic or clinical practice characteristic, with moral distress. We then conducted multivariable logistic regression to predict moral distress from all demographic and clinical practice characteristics simultaneously, except graduation year and percent clinical effort in inpatient/outpatient settings.

We quantitatively examined bivariate associations between each of the general and specific GoCC attitude and behavior items with moral distress. We dichotomized or trichotomized responses to each general and specific GoCC attitude and behavior item. We used Chi-square or Fisher exact tests, as appropriate, to assess associations with moral distress.

All analyses were conducted using SAS EG 8.3 software. Individuals with missing data on particular survey items were excluded from relevant analyses; we report the number of missing values for any variable within Table footnotes.

### Qualitative

The full qualitative team (J.A.P., M.M., A.M.L., R.S.W.) took a deductive and an inductive approach (i.e., a hybrid approach) to thematic analysis of free-text responses to the open-ended moral distress survey question. Deductive codes were identified from terms and concepts within the scientific literature; these terms and concepts served as broad *a priori* domains of potential contributors to moral distress (e.g., contributors at the individual-level, system-level, situation-level). The qualitative team identified inductive codes by reading, re-reading, and interpreting the open response data [23, 24].

Using this hybrid deductive and inductive approach, the qualitative team generated an initial set of codes. Working from these initial codes, J.A.P. and M.M. iteratively refined the codes and developed a codebook with definitions. The full qualitative team then applied the codebook to a subset of the data and finalized the codebook (see S2 Table). The full team then used the finalized codes to organize the data conceptually. J.A.P. identified key themes with quotes, and this analysis was reviewed and discussed by the rest of the team until consensus was established. Microsoft Word and Microsoft Excel were used for data management.

### Mixed methods

Following a convergent mixed-methods design, we analyzed quantitative and qualitative data concurrently [25]. Our quantitative and qualitative analytic processes are described above. In mixed methods fashion, our team integrated findings at the analytic level by comparing the quantitative results with the qualitative themes for evidence of mutual confirmation.

## Results

### Respondent demographics, clinical practice characteristics, and experience of moral distress

Out of 3,396 eligible clinicians, there were 323 respondents who opted to participate in the survey (response rate 9.5%). Respondent demographics were largely consistent with those of the eligible sample of clinicians from which they were drawn (see S3 Table), though respondents were more likely to be female (63% v. 43%). Respondents were also more likely to have a specialty in geriatrics/palliative care (26% v. 9%) and less likely to have a specialty in internal medicine/primary care/family medicine (28% v. 45%) compared to the eligible sample. This may reflect the topic of the survey (GoCCs) being of particular interest or relevance to clinicians in geriatrics/palliative care. Additional respondent characteristics included being aged 50–59 years (34%), self-identifying as white (64%), and working as an attending physician (58%). Pre-Covid, respondents spent most of their clinical time in outpatient settings (median 85%; interquartile range: 30–100%) and minimal time in inpatient settings (median 5%; interquartile range: 0–45%). Eighty-one percent of respondents [261/321 (2 respondents did not indicate level of moral distress)] experienced some level of moral distress during peak-Covid [None = 19% (60/321), Mild = 28% (89/321), Uncomfortable = 30% (98/321), Intense = 18% (59/321), Severe = 5% (15/321)]. See Table 1 for presentation of demographics and clinical practice characteristics by moral distress dichotomized as heightened (“Intense”/“Severe”) or not (“None”/”Mild”/”Uncomfortable”). See S4 Table for presentation of demographics and clinical practice characteristics by each level of moral distress.

**Table 1:** Respondent characteristics.

	Total (N = 321*) n (%)	None/Mild/Uncomfortable (N = 247) n (%)	Intense/Severe (N = 74) n (%)	P-value
**Age**				0.50^†^
20–39	42 (13)	31 (13)	11 (15)	
40–49	83 (26)	60 (24)	23 (31)	
50–59	109 (34)	85 (34)	24 (32)	
60+	87 (27)	71 (29)	16 (22)	
**Gender**				0.01^†^
Male	112 (35)	96 (39)	16 (22)	
Female	201 (63)	144 (58)	57 (77)	
**Race**				0.11^†^
White	207 (64)	165 (67)	42 (57)	
Non-White	114 (36)	82 (33)	32 (43)	
**Ethnicity**				0.62^†^
Hispanic	18 (6)	13 (5)	5 (7)	
Non-Hispanic	303 (94)	234 (95)	69 (93)	
**Role**				0.30^†^
Physician	186 (58)	147 (60)	39 (53)	
Advanced Nurse Practitioner/Physician Assistant	135 (42)	100 (40)	35 (47)	
**Specialty**				0.18^†^
Internal Medicine/Primary Care/Family Medicine	89 (28)	74 (30)	15 (20)	
Geriatrics/Palliative Care	85 (26)	68 (28)	17 (23)	
Emergency Medicine/Pulmonary Medicine/Critical Care	48 (15)	34 (14)	14 (19)	
Other^§^	99 (31)	71 (29)	28 (38)	
**Year of Graduation**, Median (IQR)				0.14^‡^
	2000 (1989–2010)	1999 (1989–2010)	2003 (1990–2011)	
**Clinical Effort**				0.12^†^
0–80%	147 (46)	119 (48)	28 (38)	
81%-100%	174 (54)	128 (52)	46 (62)	
**Clinical Setting**, Median (IQR)				0.47^‡^
Inpatient	5 (0–45)	5 (0–45)	10 (0–40)	
Outpatient	85 (30–100)	85 (30–100)	85 (40–100)	
**At least one GoCC during the pandemic** ^**||**^				0.68^†^
Yes	13 (4)	10 (4)	3 (4)	
No	68 (21)	55 (22)	13 (18)	

*Two subjects with missing Moral Distress answers were excluded for all analyses

^†^Chi-Square p-value

^‡^Kruskal-Wallis p-value

^§^Reported “Other” specialties include anticoagulation services, allergy and immunology, addiction medicine, cardiology, dermatology, endocrinology, employee health services, hematology/oncology, infectious disease, nephrology, neurology/traumatic brain injury, occupational health services, psychiatry/mental health, pain medicine, rheumatology, radiologic services, surgery, wound care, and unspecified

^||^This item had 242 missing values.

### Associations of moral distress with demographic and clinical practice characteristics

In bivariate analyses, only gender was significantly associated with experiencing heightened moral distress (Table 1). This effect held in the multivariable logistic regression model (Table 2), with significantly higher reporting of heightened moral distress by women compared to men (OR: 3.35; 95% CI, 1.53–7.37). The multivariable logistic regression also revealed significant differences in moral distress by specialty; compared to those practicing in medical subspecialties other than pulmonary/critical care (e.g., cardiology), the odds of experiencing heightened moral distress were significantly lower in geriatrics/palliative care (OR: 0.40; 95% CI, 0.18–0.87) and internal medicine/family medicine/primary care (OR: 0.46; 95% CI, 0.22–0.98).

**Table 2:** Clinician characteristics associated with moral distress (n = 302*).

	Odds Ratio^†^	95% Confidence Interval
**Gender (ref: Male)**		
Female	3.34	1.52–7.37
**Age (ref: 60+ years)**		
20–39 years	1.30	0.49–3.46
40–49 years	1.21	0.52–2.82
50–59 years	0.93	0.41–2.11
**Race (ref: white)**		
Non-white	1.22	0.63–2.36
**Ethnicity (ref: Non-Hispanic)**		
Hispanic	1.52	0.45–5.10
**Role (ref: Physician)**		
Advanced Nurse Practitioner/ Physician’s Assistant	0.93	0.48–1.79
**Specialties (ref: Other** ^**‡**^ **)**		
Internal Medicine/Primary Care/ Family Medicine	0.46	0.22–0.98
Emergency Medicine/Pulmonary Medicine/Critical Care	1.03	0.43–2.46
Geriatrics/Palliative Care	0.40	0.18–0.87
**Percent Clinical Duty (ref: 81–100%)**		
80%	0.78	0.41–1.45

*Missing responses: n = 21

^†^ Adjusting for all demographic and clinical practice characteristics except graduation year and percent clinical effort in inpatient/outpatient settings (i.e., all estimates control for all other variables in the table)

^‡^ “Other” specialties include anticoagulation services, allergy and immunology, addiction medicine, cardiology, dermatology, endocrinology, employee health services, hematology/oncology, infectious disease, nephrology, neurology/traumatic brain injury, occupational health services, psychiatry/mental health, pain medicine, rheumatology, radiologic services, surgery, wound care, and unspecified.

### Associations of moral distress with goals of care conversation attitudes and behaviors during peak-Covid

Compared to clinicians with lower levels of moral distress, those with heightened moral distress were more likely to agree that, during peak-Covid-19, providing a patient with suspected or confirmed Covid with a specific recommendation about LST: “further burdens the patient” (34% vs. 15%, p = 0.0002), “unduly influences the patient’s decision” (36% vs. 20%, p = 0.003), and “places too great a burden on the provider” (31% vs. 18%, p = 0.01). Clinicians with heightened moral distress were more likely to respond that making independent LST decisions themselves and subsequently informing patients of that decision was ethically appropriate during peak-Covid-19 (22% vs. 11%, p = 0.02). Additional results from the bivariate analyses are shown in S5 Table.

### Qualitative findings

There were 191 clinicians who provided responses to the open-ended item about circumstances contributing to their peak-Covid moral distress [59% (191/323) of the total survey sample and 73% (191/261) of the subsample indicating any level of moral distress]. The subsample of respondents to the open-ended item was characterized as 68% white, 65% female, 35% aged 50–59 years, 60% worked as an attending physician, and 28% worked in geriatrics or palliative care. Before the pandemic, respondents spent a median of 80% (interquartile range: 30–100%) of their clinical time in the outpatient setting and a median of 10% (interquartile range: 0–50%) of their clinical time in the inpatient setting. (See Table 3).

**Table 3:** Characteristics of respondents to open-ended item about moral distress.

	Total (N = 191*) n (%)	Mild/Uncomfortable (N = 140) n (%)	Intense/Severe (N = 51) n (%)	P-value
**Age**				0.49^†^
20–39	23 (12)	16 (11)	7 (14)	
40–49	55 (29)	37 (26)	18 (35)	
50–59	67 (35)	50 (36)	17 (33)	
60+	46 (24)	37 (26)	9 (18)	
**Gender**				0.11^†^
Male	62 (32)	50 (36)	12 (24)	
Female	125 (65)	86 (61)	39 (76)	
**Race**				0.61^†^
White	129 (68)	96 (69)	33 (65)	
Non-White	62 (32)	44 (31)	18 (35)	
**Ethnicity**				0.76^†^
Hispanic	13 (7)	10 (7)	3 (6)	
Non-Hispanic	178 (93)	130 (93)	48 (94)	
**Role**				0.07^†^
Physician	114 (60)	89 (64)	25 (49)	
Advanced Nurse Practitioner/Physician Assistant	77 (40)	51 (36)	26 (51)	
**Specialty**				0.07^†^
Internal Medicine/Primary Care/Family Medicine	48 (25)	42 (30)	6 (12)	
Geriatrics/Palliative Care	53 (28)	38 (27)	15 (29)	
Emergency Medicine/Pulmonary Medicine/Critical Care	29 (15)	20 (14)	9 (18)	
Other^§^	61 (32)	40 (29)	21 (41)	
**Yr. of Graduation** Median (IQR)				0.05^‡^
	2001 (1990–2010)	1999 (1990–2009)	2004 (1998–2011)	
**Clinical Effort**				0.15^†^
0–80%	95 (50)	74 (53)	21 (41)	
81%-100%	96 (50)	66 (47)	30 (59)	
**Clinical Setting** Median (IQR)				0.64^‡^
Inpatient	10 (0–50)	10 (0–50)	15 (0–50)	
Outpatient	80 (30–100)	80 (30–100)	80 (30–100)	
**At least one GoCC during the pandemic** ^||^				0.85^†^
Yes	6 (3)	4 (3)	2 (4)	
No	34 (18)	24 (17)	10 (20)	

*Two subjects with missing Moral Distress answers were excluded for all analyses

^†^Chi-Square

p-value

^‡^Wallis p-value

^§^“Other” specialties include anticoagulation services, allergy and immunology, addiction medicine, cardiology, dermatology, endocrinology, employee health services, hematology /oncology, infectious disease, nephrology, neurology/traumatic brain injury, occupational health services, psychiatry/mental health, pain medicine, rheumatology, radiologic services, surgery, wound care, and unspecified; ^||^This item had 242 missing values.

We identified five qualitative themes about contributors to clinicians’ moral distress early in the pandemic. Theme #1, *Clinical uncertainty surrounding Covid-19*, highlighted how the clinical “unknowns” about Covid-19 early in the pandemic led to clinicians feeling that they were not adequately meeting patients’ treatment and informational needs, an abrogation of their professional ethos. Theme #2, *Anticipatory actions*, pointed to how policies and clinician behaviors that pre-empted the need to triage resources, at times needlessly, led to a sense of transgressing patients’ treatment needs. Theme #3, *Unprecedented restrictions on patient visitation*, related to clinicians’ role as enforcers of restricted patient visitation; this role led to patients being isolated and dying alone which fostered a sense of moral breach and a situation that was “horrific” and “the biggest tragedy of the entire pandemic.” Theme #4, *Personal risk to clinicians*, underscored how the risk of contracting Covid-19 while treating patients caused clinicians to fear for their own and their families’ safety; thus, they felt caught between two moral obligations: a personal obligation (to family and self) and a professional one (to patients). Theme #5, *Resource shortages*, described how scarcity in supplies, personnel, time, and financial means lead to resource allocation decisions that made clinicians feel morally compromised in their efforts to address patients’ needs. Exemplary quotes by theme are seen in Table 4.

**Table 4:** Qualitative themes with exemplary quotes.

Theme	Exemplary Quotes
#1: Clinical uncertainty surrounding COVID-19	*“The lack of data on an effective therapy made me most uncomfortable*. *I am an intensivist and decisions of life sustaining measures is a usual thing in our line of work*.* *.* *. *I had several years to dissect out how to be morally consistent with myself…[previously] We could answer patients and surrogate’s [sic] question with a level of certainty*. *During Peak COVID this certainty was not there*. *We simply did not know*. *We relied on our understanding of pulmonary physiology and ARDS [acute respiratory distress syndrome] and it was OK but the virus had a lot of dark twists under its sleeve and their [sic] was a level of guilt and a level of feeling inadequate*.*” (ID #161)* *“With the guidelines changing so quickly*, *I didn’t feel like we knew what we were doing and that was very uncomfortable to share with patients and families*. *They come to us for help*, *because we are the experts*, *but we just didn’t know*.* *.* *.*” (ID #198)*
#2: Anticipatory actions	*“…[age cut-offs for allocating resources] never in fact materialized but I believe did harm patients in leading to undertreatment*, *marginalization and ageism*.*” (ID #16)* *“Limiting services due to lack of supplies or equipment is very distressing*. *In extraordinary times of crises*, *we have no other choice*. *However*, *my feeling was that the issue became so politicized and the handling of the crises was done so poorly that “critical shortage” decisions were unnecessarily forced upon us—leading to moral distress*.*” (ID# 130)*
#3: Unprecedented restrictions on patient visitation	*“Being the gatekeeper for restricted family visitation*, *i*.*e*., *having to tell or explain to family members they can’t visit at the end of life and similar circumstances*, *(even when Covid was not a factor) was the absolute worst thing I’ve had to do in my 30-year career in mostly end-of-life care*. *It has made me bitter*, *resentful*, *frequently tearful*, *and regularly reconsider other career choices and options*. *I have watched these restrictions*, *and their enforcement*, *take a serious mental health toll on much of my staff…It is the one part of Covid I don’t think I will ever fully recover from*.*”* (ID #246) *“I think the biggest ethical challenge has been not allowing family members to be with their loved ones during hospitalizations*, *creating trauma for both those involved and providers*”. (ID #199)
#4: Personal risk to clinician	*“I have high risk family members*, *and concern about seeing patients face to face*. *Family was very concerned*, *I lived in other rooms and limited family time*. *My manager was VERY supportive of any decision I made regarding face-to-face visits*, *however I felt an overall push/stress from the HCS to see patients*, *volunteer for covid exposure assignments and overall sense of not fulfilling my responsibility if I limited my exposures*. *I had a great deal of personal conflict*, *feeling like I was letting down coworkers*.*” (ID #8)* “*When personal risk [to family and children] gets included into the discussion and situation*, *the metrics for my willingness to provide care were unexpectedly brought into question*.*” (ID #24)*
#5: Resource shortages	*“I [sic] felt like i [sic] was on a lifeboat when the titanic [sic] was sinking*. *I was telling patients not to come to the hospital because we did not have the PPE*, *we did not have covid [sic] testing*. *Like saying*, *don’t jump into [sic] this lifeboat or we will all sink*.*” (ID #53)* *“There was concern that we would be unable to provide care that patients would ordinarily expect to receive—-because of the impact of COVID-19 demands on resources*, *coupled with inadequate preparation*. *It was like breaking a promise to patients*.*” (ID #190)*

### Mixed methods findings

Two sets of quantitative results and qualitative themes were consistent with one another. First, we found that heightened versus lower moral distress was significantly associated with considering it appropriate to limit LST for Covid-19 patients due to personal risks to clinicians (30% vs. 19%, p = 0.04). This quantitative result is consistent with qualitative Theme #4 (Personal risk to clinicians). Second, we found that heightened versus lower moral distress was significantly associated with considering it appropriate to limit LST for Covid-19 patients due to limited resource availability (62% vs. 35%, p< 0.0001). This quantitative result is consistent with qualitative Theme #5 (Resource shortages).

## Discussion

In our mixed methods study, the majority of VA clinician respondents experienced at least mild moral distress during early Covid-19. Survey responses also shed light on correlates (i.e., gender and specialty) and contributors (i.e., clinical uncertainty surrounding Covid-19; anticipatory actions; unprecedented restrictions on patient visitation; personal risk to clinicians; and resource shortages) to such moral distress.

We found that female clinicians reported higher levels of Covid-related moral distress than male clinicians, which aligns with or extends findings from other studies showing that gender is associated with the likelihood of moral distress. While this association only emerged after controlling for other demographic and practice characteristics, it is corroborated by the literature on the topic both pre-pandemic and early pandemic. Pre-pandemic, results from a small survey of critical care nurses in the US (N = 31) were suggestive of higher average moral distress scores among female versus male subjects [26]. Early pandemic, a larger study of 1,606 hospital-based nurses and physicians in a Western Norwegian survey also demonstrated higher levels of moral distress in female respondents [27]. Similar to our study, the Norwegian survey was conducted in a large healthcare system (inclusive of as many as 50 institutions) across several specialties (medical, surgical, anesthesia or internal care medicine, psychiatry and addiction medicine). Combined with our findings, these consistent results may indicate a need to address moral distress through interventions tailored to gender-specific experiences, regardless of whether clinicians are those who conduct GoCCs (as in our study).

We also found that clinical specialty was associated with the likelihood of experiencing moral distress during the early pandemic, a finding partially corroborated by existing literature. In our study, after controlling for demographic and practice characteristics, clinicians practicing in geriatrics/palliative care and internal medicine/primary care/family medicine were less likely to have heightened moral distress than those in other specialties. In prior research there are conflicting findings as to whether clinical specialty is associated with moral harm to clinicians. Miljeteig et al’s Norwegian survey (mentioned above) demonstrated cross-specialty differences: clinicians practicing in medical, surgical, anesthesia, or intensive care medicine were less likely to have heightened moral distress than those in psychiatry/addiction medicine [27]. In contrast, in a U.S. survey of 595 healthcare workers in outpatient, inpatient, and emergency department settings, moral injury symptomatology did not significantly differ by specialty [28]. The contrasting findings of Rushton et al.’s U.S. survey compared to Miljeteig et al’s Norwegian survey and ours could reflect the different constructs being examined (moral injury versus moral distress); yet, all three studies may suggest a similar conclusion. That is, the Norwegian survey and ours support the possibility of a “novice effect” on the likelihood of moral distress in early Covid-19 [27]. Clinicians who are "novices” in a particular healthcare context (e.g., psychiatrists working with Covid-19 patients through redeployment) were more likely to experience moral distress than clinicians with greater experience in the same context [27]. This same principle may have been seen among our study sample, whereby clinicians who were redeployed to unfamiliar settings or who had less prior exposure to end-of-life decision-making processes (e.g., specialties other than geriatrics/palliative care or internal medicine/primary care/family medicine) experienced higher levels of moral distress. Even Rushton et al.’s findings suggest a novice effect; despite there being no specialty differences in their study; that is, clinicians having worked fewer years in their profession had higher levels of moral injury [28]. Thus, these three sets of results might all underscore the need for customized interventions that support clinicians in situations new to them during healthcare crises.

New insights from our qualitative themes include two contributors to clinician moral distress (Themes #1 and #2) that have not previously been identified in the Covid-19 literature. Our Theme #1 (*Clinical uncertainty surrounding Covid-19*) suggests how the “unknowns” of a novel disease may create clinical situations where clinicians feel unable to honor their own ethical beliefs, whether due to lack of knowledge about the disease or because of limited experience communicating with patients about the novel entity. Our Theme #2 (*Anticipatory actions*) may reflect a natural corollary of Theme #1. That is, clinical uncertainty surrounding a new and unknown disease may result in healthcare stakeholders imagining and acting upon worst-case scenarios (e.g., by rationing resources pre-emptively with unintended negative consequences). Future studies are needed to operationalize and validate these two contributors as well as to assess their association with clinician moral distress. If indeed validated, these two novel constructs suggest the need to develop consultative services within healthcare institutions to guide stakeholders in navigating fluctuating and unpredictable clinical circumstances.

Meanwhile, one of our qualitative themes (Theme #3: *Unprecedented restrictions on patient visitation)* has been noted in extant Covid-19-based research. As reflected in this theme, our surveyed clinicians often described how their moral distress derived from a role as gatekeeper of patient visitation restrictions, restrictions which could then lead to patients dying alone. One study on visitation restrictions during Covid-19 consisted of ten interviews with intensive care nurses in Sweden [29]. Nurses were asked not about the impact that these restrictions had on them personally but rather about the kinds of clinical challenges resulting from them. Nurses mentioned it was a challenge to know when to make exceptions to the restrictions, a construct which was not specifically mentioned in our work but might indeed be part of the gatekeeper role that our respondents described. Our Theme #3 particularly resonated with another qualitative study that interviewed 45 Canadian intensive care and medical ward clinicians caring for dying patients [30]. Similar to our work, clinician moral distress emanated from vacillating visitation rules and inconsistently applied exceptions to rules, especially when these rules and exceptions denied patients and families the opportunity to say “goodbye” to a dying patient. Finally, in Rushton et al.’s U.S.-based survey, as described above, healthcare workers who scored higher on symptoms of moral injury also endorsed a statement that Covid-19’s extreme visitation restrictions altered the process of LST decisions by curbing family involvement [28]. Our study and the three studies to which we compare ours examine a variety of constructs (moral distress or otherwise) but universally found the unprecedented visitation restrictions of the pandemic to be professionally challenging. This body of literature emphasizes the need to implement interventions in future healthcare crises that maintain family involvement in patient care, especially at end-of-life, and that clarify–or even obviate–clinicians’ gatekeeping role regarding patient visitation.

Our quantitative and qualitative findings aligned with one another in identifying the influence of Covid-related *Personal risk to clinicians* (Theme #4) and *Resource shortages* (Theme #5) on clinicians’ moral distress. These two factors are documented as contributing to anxiety [31] and moral injury [28, 32] within an array of healthcare disciplines and settings. One U.S. study conducted eight listening sessions with physicians, residents, fellows, nurses, and advanced practice practitioners (total N = 69) [31]. Participants described the anxiety experienced at the onset of Covid-19 in relation to several factors, including the risk of spreading infection to their families and the accessibility of personal protective equipment; these factors align with our Theme #4 and Theme #5, respectively. Our study builds upon this study by extending these findings beyond the context of Covid-related anxiety to the context of Covid-related moral distress.

Two studies examined moral injury in clinicians early in the pandemic with findings also echoing our Themes #4 and #5. One qualitatively analyzed open-ended survey responses from 1,334 physicians, nurses, advanced practice practitioners, and chaplains largely working in the U.S. [32]. Respondents described the moral injury-related stressors they experienced as including the risk of contagion and shortages of staff and personal protective equipment. Rushton et al, described above, found moral injury symptoms were correlated with practicing clinically with limited resources and when there were personal concerns of occupational acquisition of Covid-19 [28]. We build on this existing knowledge by focusing specifically on moral distress, a prelude to the more severe and less tractable experience of moral injury. Accordingly, our study offers ideas for intervening when clinician moral distress is present and before it advances to moral injury.

As alluded to above, ours and others’ findings point to possible leverage points for future intervention. Personal and professional characteristics (gender, specialty) suggest the potential for providing tailored emotional support to clinicians during healthcare crises, including clinicians who have to assume clinical duties that are new to them. Support networks could be established throughout the organization: from the level of colleagues to supervisors to organizational leaders [4, 12, 33]. Support could also be provided through resources like crisis hotlines, websites, peer support programs, and employer-based counseling services [34]. Tailored training and educational opportunities [30, 35] should also be offered to clinicians who are facing high risk for moral distress; indeed, an integrative review of moral distress interventions found those that were educational to be most successful [36]. Training and education could instruct clinicians on, for example, personal resources (mindfulness practices) or professional skills (communication approaches with patients and families, ethical decision-making) [8, 34]

The influences of both clinical uncertainty surrounding Covid and anticipatory actions on moral distress point to the need for flexible and situation-specific interventions. One such intervention would be for healthcare facilities to provide clinicians with not just ethics consultation services (as is often done in the hospital setting) but also moral distress consultation services. In a pre-pandemic prototype of this hybrid model, the combined services were staffed with consultants of multiple disciplines, and clinicians could page an on-call consultant for triage to appropriate services [37]. Such a model aims to improve healthcare quality by addressing unit-level and system-level contributors to clinician moral distress [37]. It also has the potential to address these contributors flexibly through consultative dialogue.

The field has called for additional interventions to address contributors to COVID-19-based moral distress (visitation restrictions, personal risk to clinicians, resource constraints) that both we and other studies have repeatedly identified. First, the impact of visitation restrictions on clinician moral distress reinforced the value of video conferencing [28–30, 38], a communication modality already used in VA for patient care which could be even more broadly implemented across healthcare systems. This contributor’s impact also led to a call for clear policies, for centralized and standardized arbitration of exceptions to visitation restrictions, and for excusing clinicians from arbitration decision-making so as not to impair relationships with their patients [38]. Second, personal risk to clinicians as a contributor highlights the need for infection control personnel to field clinicians’ questions and concerns and to share the most up-to-date information on best infection control practices [3]. Clinicians who become infected should receive enhanced support from their occupational health department as well as augmented employment benefits (e.g., sick leave distinct from already allocated paid time off) [31, 33]. Third, resource constraints as a contributor highlight the need for healthcare facility leaders and infection control personnel to develop and enact transparent guidelines for the use/reuse and rationing of personal protective equipment and to communicate them directly to clinicians both initially and as they may change [3].

There are limitations to this study. First, we had a relatively low response rate of 9.5%, though well-distributed variation in respondent characteristics may have minimized the potential for non-response bias. As with all retrospective surveys, there is potential for inaccuracies in recall since respondents reflect on attitudes and behaviors in the past (roughly a year prior in this instance); however, there is no reason to think this would create a bias in one direction or another. Respondents were asked to define peak-Covid based on local factors and thus might not have reflected upon the exact same dates across sites; however, this flexibility allowed us to examine each respondent’s subjective experiences of peak-Covid.

Our survey instrument had limitations as well. Full psychometric properties were not assessed for the two pre-existing instruments (upon which our instrument was based) nor our own survey instrument, though all three underwent pilot testing after initial development as well as iterative content revision. Also, we used a modified single item to assess moral distress and dichotomized it to focus on heightened moral distress for analyses. Future work attempting to replicate and extend these findings would benefit from using a multi-item validated scale [39, 40] and exploring predictors of more moderate levels of moral distress as well.

Potential limitations also exist regarding the generalizability of our findings. First, respondents in our survey were predominately white, female, and over the age of 50, though this demographic pattern is consistent with other survey studies of VA-based clinicians [41–44], and the characteristics of our respondents were fairly comparable to those of the eligible population from which they were drawn (see S3 Table), (though female clinicians and those in geriatric/palliative care were more common among respondents). There is also the possibility that our findings are not generalizable or transferable to clinicians who work in settings outside of the VA.

## Conclusion

This mixed methods study highlights the notably prevalent moral distress experienced by VA clinicians early in the pandemic as well as contributors to that moral distress (i.e., gender; specialty; clinical uncertainty surrounding Covid-19; anticipatory actions; unprecedented restrictions on patient visitation; personal risk to clinicians; and resource shortages). The role of each contributor indicates possible leverage points for intervention during future healthcare crises and, perhaps, during everyday care (for example, care related to end-of-life decision-making processes). Possible intervention include: 1) providing support services and training and educational opportunities tailored to clinicians’ personal characteristics; 2) establishing moral distress committees within healthcare institutions to adapt nimbly to fluctuating and unpredictable clinical demands; 3) developing clear arbitration policies for changes in patient visitation rules that do not rely on clinicians to serve as arbiters; 4) increasing involvement of infection control personnel on the front lines and expanding employee healthcare benefits and access to occupational health; and 5) ensuring transparent and frequent communication about resource shortage management. Not all of these intervention ideas are novel nor feasible within all healthcare institutions. More post-hoc consideration of the moral distress experience during Covid-19 may be needed to generate additional ideas. With a broad array of ideas at hand–both those described here and future ones—healthcare systems could better mitigate the deleterious impact of clinicians’ moral distress on clinicians’ well-being, patients’ well-being, and healthcare system function.

## Supporting information

S1 Checklist STROBE statement—checklist of items that should be included in reports of observational studies. (DOCX)

S1 File Survey instrument. (DOCX)

S1 Table Original sources of individual survey items. (DOCX)

S2 Table Codebook for qualitative analysis. (DOCX)

S3 Table Demographic information of study sample vs. total eligible sample. (DOCX)

S4 Table Respondent characteristics (Total and comparative by each level of moral distress). (RTF)

S5 Table Attitudes and behaviors related to goals of care conversations. (DOCX)
