# Supplementary material for: Addressing clinician moral distress: Implications from a mixed methods evaluation during Covid-19
Source: PLoS One. 2023 Sep 15;18(9):e0291542. doi: 10.1371/journal.pone.0291542 (PMC10503769; doi:10.1371/journal.pone.0291542)
Supplement: S1 Checklist — (DOCX) [file pone.0291542.s001.docx]

STROBE Statement—checklist of items that should be included in reports of observational studies

|  | Item No. | Recommendation | Page  No. | Relevant text from manuscript |
| --- | --- | --- | --- | --- |
| **Title and abstract** | 1 | (*a*) Indicate the study’s design with a commonly used term in the title or the abstract | 1 | Title: Addressing clinician moral distress: implications from a mixed methods evaluation during Covid-19 |
|  |  | (*b*) Provide in the abstract an informative and balanced summary of what was done and what was found | 3-4 | **Background:** The Covid-19 pandemic resulted in moral distress, burnout, and turnover among clinicians.  **Objective:** This convergent mixed methods study examined prevalence of and contributors to Covid-related moral distress among Veterans Health Administration (VA) clinicians.  **Design:** Multi-site cross-sectional survey in April 2021 with inductive thematic analysis and bivariate and multivariable regression analyses to understand factors associated with heightened moral distress.  **Setting:** Twenty VA Medical Centers evenly distributed across the nation’s four census regions with the greatest number of cumulative Covid-19 cases early in the pandemic.  **Participants:** VA clinicians with professional jurisdiction to place life-sustaining treatment orders practicing in select specialties (n=3,398).  **Measurements:** Respondents’ demographics, clinical practice characteristics, attitudes and behaviors related to goals of care conversations, intensity of moral distress during “peak-Covid,” and an open-ended item to describe contributing circumstances for respondents indicating any moral distress.  **Results:** There were 323 respondents (9.5% adjusted response rate). Most (81%) reported at least some moral distress during peak-Covid. Five qualitative themes emerged as moral distress contributors: 1) patient visitation restrictions, 2) anticipatory actions, 3) clinical uncertainty related to Covid, 4) resource shortages, and 5) personal risk of contracting Covid. Quantitative analyses corroborated these last two themes. In a multivariable logistic regression, factors associated with moral distress were female gender (OR 3.35; 95% CI 1.53-7.37) and practicing in geriatrics/palliative care (OR 0.40; 95% CI 0.18-0.87) and internal medicine/family medicine/primary care (OR 0.46; 95% CI 0.22-0.98) compared to medical subspecialties.  **Conclusion:** Moral distress was prevalent early in the pandemic. Individual-, system-, and situation-level contributors indicate leverage points for future intervention to mitigate moral distress. |
| Introduction | | | |  |
| Background/rationale | 2 | Explain the scientific background and rationale for the investigation being reported | 4 | Moral distress within healthcare occurs when internal or external conditions cause clinicians to provide care in ways that they feel have transgressed their ethical beliefs. While moral distress is sometimes used interchangeably with “moral injury,” it is a separate construct that is a precursor to the more severe experience of moral injury [1]. The onset of the Covid-19 pandemic exacerbated clinician moral distress in many healthcare systems [2, 3]. Some pandemic-related contributors to moral distress include unprecedented levels of restrictions on patient visitation, personal risk to clinicians, and physical resource shortages [2, 4].  Clinician moral distress can have multiple negative impacts, including harm to clinicians’ physical well-being (e.g., by leading to appetite loss, gastrointestinal symptoms, migraines) [5] and emotional well-being (e.g., by leading to anger, sadness, guilt, shame) [3, 6, 7]. Such harms can translate to impaired clinical practice, endangering the quality of patient care [8] and, likely, patient health outcomes. Moral distress is also associated with clinician burnout and turnover [8-10], which can undermine healthcare system functioning. Even pre-pandemic, the challenges resulting from clinician moral distress have been noted internationally across professional disciplines and healthcare settings. The pandemic’s global presence only threatens to further amplify these challenges.  To date, there is minimal published research on Covid-19-related moral distress among clinicians within the Veterans Health Administration (VA), the largest nationally integrated healthcare system in the U.S. Understanding the magnitude of and contributors to VA clinicians’ moral distress can provide important information to both VA and other healthcare settings about areas for intervention during routine care and in advance of future national healthcare crises. Preventing clinician moral distress or intervening when it does occur could minimize its negative impacts. |
| Objectives | 3 | State specific objectives, including any prespecified hypotheses | 4-5 | Thus, we used a convergent mixed methods approach with VA survey data collected at the onset of the Covid-19 pandemic to investigate the prevalence of and contributing factors to VA clinicians’ Covid-related moral distress. |
| Methods | | | |  |
| Study design | 4 | Present key elements of study design early in the paper | 4-5 | Thus, we used a convergent mixed methods approach with VA survey data collected at the onset of the Covid-19 pandemic to investigate the prevalence of and contributing factors to VA clinicians’ Covid-related moral distress. |
| Setting | 5 | Describe the setting, locations, and relevant dates, including periods of recruitment, exposure, follow-up, and data collection | 6 | (Lines 95-98): We administered a cross-sectional survey to healthcare clinicians from 20 VA Medical Centers. The centers were evenly distributed across the nation’s four census regions and were selected for having experienced the greatest number of cumulative Covid-19 cases between March 2020 and October 2020.  (Lines 111-112): We invited potential respondents by email to complete the survey electronically between March and April 2021. |
| Participants | 6 | (*a*) *Cohort study*—Give the eligibility criteria, and the sources and methods of selection of participants. Describe methods of follow-up  *Case-control study*—Give the eligibility criteria, and the sources and methods of case ascertainment and control selection. Give the rationale for the choice of cases and controls  *Cross-sectional study*—Give the eligibility criteria, and the sources and methods of selection of participants | 6 | We identified eligible clinicians from the VA Corporate Data Warehouse based on three criteria: 1) working as licensed independent clinicians (e.g., physicians, nurse practitioners, physician assistants) with the authority to place life-sustaining treatment (LST) orders in the electronic health record; 2) providing direct patient care in inpatient, outpatient, or long-term care settings; and 3) practicing in the discipline of internal medicine or its relevant subspecialties (e.g., cardiology, geriatrics, palliative care medicine, pulmonary and critical care), emergency medicine, family medicine, neurology, or surgery. |
|  |  | (*b*) *Cohort study*—For matched studies, give matching criteria and number of exposed and unexposed  *Case-control study*—For matched studies, give matching criteria and the number of controls per case | N/A | N/A |
| Variables | 7 | Clearly define all outcomes, exposures, predictors, potential confounders, and effect modifiers. Give diagnostic criteria, if applicable | 7-9 | We developed a survey by adapting two pre-existing instruments which focused on physicians’ attitudes and behaviors regarding goals of care conversations (GoCCs) [11, 12]. Details on survey development are provided elsewhere [13]. (See Appendix Table 1 for survey instrument.)  Primary outcome: Moral Distress – The survey included two items related to experiences of moral distress, which we defined as: “when professionals are unable to carry out what they believe to be ethically appropriate actions because of internal (e.g. fear of repercussions; self-doubt) or external (e.g. lack of support, hierarchies of healthcare system) constraints” [14]. First, a closed-ended item asked the respondent to rate the intensity of overall level of moral distress during peak-Covid, with response options spanning a 5-point Likert scale (i.e., “None”, “Mild”, “Uncomfortable”, “Intense”, “Severe”). Any respondent who selected a response option other than “None” was presented with an optional open-ended item: “Can you tell us more about the circumstances that may have contributed to these feelings?”  General Attitudes about Goals of Care Conversation during Peak-Covid-19 – Respondents were instructed to identify a period of “peak-Covid-19,” defined as the period when workload, work hours, clinical demand, and Covid-19 cases were highest. Clinicians were then asked a series of questions about their attitudes related to GoCCs during peak-Covid-19. The survey had two items regarding general attitudes about LST recommendations during GoCCs, including perceived appropriateness (using a 4-point scale from “Very appropriate” to “Very inappropriate”) and comfort with giving LST recommendations (using a 4-point scale from “Very comfortable” to “Very uncomfortable”). Next, there were six items about the general ethics of providing specific LST recommendations (e.g., it unduly influences patients, it is only appropriate if wanted); response options were presented on a 4-point scale from “Disagree strongly” to “Agree strongly.” There were then nine items about the ethical appropriateness of using specific dialogue techniques during GoCCs (e.g., use vivid imagery, discuss small chance of recovery); response options were presented on a 4-point scale from “Definitely not appropriate” to “Definitely appropriate.”  Specific Attitudes and Behaviors in Goals of Care Conversations during peak-Covid – The survey contained 12 items concerning Covid-specific GoCC attitudes and behaviors. Clinicians reported how often they asked patients with Covid if they wanted a recommendation about LST (e.g., cardiopulmonary resuscitation) decisions (5-point scale ranging from “Never” to “Always”). They also rated the perceived appropriateness of limiting a patient’s LST options (on a 4-point scale from “Definitely not appropriate” to “Definitely appropriate” because of the risk it poses to healthcare providers and because of limited resources for other patients, as well as how concerned they were (on a 5-point scale from “Not at all” to “Extremely”) about resource availability at their facility. Other survey items assessed comfort with prognosticating about whether patients will have outcomes consistent with their goals and values after respiratory failure for both patients with and without Covid (on a 4-point scale from “Very comfortable” to “Very uncomfortable”). Finally, a series of items addressed quality of GoCCs during peak-Covid, including how frequently clinicians felt confident that they provided patients with adequate information to make a fully informed decision (on a 5-point scale from “Never” to “Always”), whether they perceived the quality of their GoCCs to be better or worse compared to pre-pandemic, and the perceived impact of two specific factors on GoCC quality (i.e., restrictions on family/support presence and communicating over telephone). They also reported whether they had at least one GoCC during peak-Covid.  Demographic and Clinical Practice Characteristics – The survey assessed several demographic (i.e., age, gender, race, ethnicity) and clinical practice characteristics [clinical role (i.e., fellow, resident, nurse practitioner or advanced practice nurse, and physician assistant), specialty (i.e., anesthesia, internal medicine, neurology, surgery, pulmonary medicine / critical care, cardiology, geriatrics, palliative care, emergency medicine, and other), year of graduation, percent clinical effort, and percent of clinical effort in inpatient and outpatient settings]. |
| Data sources/ measurement | 8* | For each variable of interest, give sources of data and details of methods of assessment (measurement). Describe comparability of assessment methods if there is more than one group | 7-9 | We developed a survey by adapting two pre-existing instruments which focused on physicians’ attitudes and behaviors regarding goals of care conversations (GoCCs) [11, 12]. Details on survey development are provided elsewhere [13]. (See Appendix Table 1 for survey instrument.)  Primary outcome: Moral Distress – The survey included two items related to experiences of moral distress, which we defined as: “when professionals are unable to carry out what they believe to be ethically appropriate actions because of internal (e.g. fear of repercussions; self-doubt) or external (e.g. lack of support, hierarchies of healthcare system) constraints” [14]. First, a closed-ended item asked the respondent to rate the intensity of overall level of moral distress during peak-Covid, with response options spanning a 5-point Likert scale (i.e., “None”, “Mild”, “Uncomfortable”, “Intense”, “Severe”). Any respondent who selected a response option other than “None” was presented with an optional open-ended item: “Can you tell us more about the circumstances that may have contributed to these feelings?”  General Attitudes about Goals of Care Conversation during Peak-Covid-19 – Respondents were instructed to identify a period of “peak-Covid-19,” defined as the period when workload, work hours, clinical demand, and Covid-19 cases were highest. Clinicians were then asked a series of questions about their attitudes related to GoCCs during peak-Covid-19. The survey had two items regarding general attitudes about LST recommendations during GoCCs, including perceived appropriateness (using a 4-point scale from “Very appropriate” to “Very inappropriate”) and comfort with giving LST recommendations (using a 4-point scale from “Very comfortable” to “Very uncomfortable”). Next, there were six items about the general ethics of providing specific LST recommendations (e.g., it unduly influences patients, it is only appropriate if wanted); response options were presented on a 4-point scale from “Disagree strongly” to “Agree strongly.” There were then nine items about the ethical appropriateness of using specific dialogue techniques during GoCCs (e.g., use vivid imagery, discuss small chance of recovery); response options were presented on a 4-point scale from “Definitely not appropriate” to “Definitely appropriate.”  Specific Attitudes and Behaviors in Goals of Care Conversations during peak-Covid – The survey contained 12 items concerning Covid-specific GoCC attitudes and behaviors. Clinicians reported how often they asked patients with Covid if they wanted a recommendation about LST (e.g., cardiopulmonary resuscitation) decisions (5-point scale ranging from “Never” to “Always”). They also rated the perceived appropriateness of limiting a patient’s LST options (on a 4-point scale from “Definitely not appropriate” to “Definitely appropriate” because of the risk it poses to healthcare providers and because of limited resources for other patients, as well as how concerned they were (on a 5-point scale from “Not at all” to “Extremely”) about resource availability at their facility. Other survey items assessed comfort with prognosticating about whether patients will have outcomes consistent with their goals and values after respiratory failure for both patients with and without Covid (on a 4-point scale from “Very comfortable” to “Very uncomfortable”). Finally, a series of items addressed quality of GoCCs during peak-Covid, including how frequently clinicians felt confident that they provided patients with adequate information to make a fully informed decision (on a 5-point scale from “Never” to “Always”), whether they perceived the quality of their GoCCs to be better or worse compared to pre-pandemic, and the perceived impact of two specific factors on GoCC quality (i.e., restrictions on family/support presence and communicating over telephone). They also reported whether they had at least one GoCC during peak-Covid.  Demographic and Clinical Practice Characteristics – The survey assessed several demographic (i.e., age, gender, race, ethnicity) and clinical practice characteristics [clinical role (i.e., fellow, resident, nurse practitioner or advanced practice nurse, and physician assistant), specialty (i.e., anesthesia, internal medicine, neurology, surgery, pulmonary medicine / critical care, cardiology, geriatrics, palliative care, emergency medicine, and other), year of graduation, percent clinical effort, and percent of clinical effort in inpatient and outpatient settings]. |
| Bias | 9 | Describe any efforts to address potential sources of bias | 10 | The qualitative team (J.A.P., M.M., A.M.L., R.S.W.) applied hybrid deductive/inductive thematic analysis to the free-text responses of the open-ended moral distress item. The four qualitative team members successively reviewed the full dataset to generate initial codes. Working from the initial codes, J.A.P. and M.M. iteratively developed a codebook containing codes and definitions; codebook development was deductively informed by and organized according to broad *a priori* domains of potential contributors to moral distress (e.g., contributors at the individual-level, system-level, situation-level). The full team successively assigned finalized codes (multiple codes when appropriate) to each free-text response. J.A.P. reviewed the coded data and identified emergent themes which were reviewed by rest of the team to establish consensus. |
| Study size | 10 | Explain how the study size was arrived at | 11 | Out of 3,398 eligible clinicians, there were 323 respondents who opted to participate in the survey (response rate 9.5%). |

Continued on next page

| Quantitative variables | 11 | Explain how quantitative variables were handled in the analyses. If applicable, describe which groupings were chosen and why | 7-9 | Primary outcome: Moral Distress – The survey included two items related to experiences of moral distress, which we defined as: “when professionals are unable to carry out what they believe to be ethically appropriate actions because of internal (e.g. fear of repercussions; self-doubt) or external (e.g. lack of support, hierarchies of healthcare system) constraints” [14]. First, a closed-ended item asked the respondent to rate the intensity of overall level of moral distress during peak-Covid, with response options spanning a 5-point Likert scale (i.e., “None”, “Mild”, “Uncomfortable”, “Intense”, “Severe”). Any respondent who selected a response option other than “None” was presented with an optional open-ended item: “Can you tell us more about the circumstances that may have contributed to these feelings?”  General Attitudes about Goals of Care Conversation during Peak-Covid-19 – Respondents were instructed to identify a period of “peak-Covid-19,” defined as the period when workload, work hours, clinical demand, and Covid-19 cases were highest. Clinicians were then asked a series of questions about their attitudes related to GoCCs during peak-Covid-19. The survey had two items regarding general attitudes about LST recommendations during GoCCs, including perceived appropriateness (using a 4-point scale from “Very appropriate” to “Very inappropriate”) and comfort with giving LST recommendations (using a 4-point scale from “Very comfortable” to “Very uncomfortable”). Next, there were six items about the general ethics of providing specific LST recommendations (e.g., it unduly influences patients, it is only appropriate if wanted); response options were presented on a 4-point scale from “Disagree strongly” to “Agree strongly.” There were then nine items about the ethical appropriateness of using specific dialogue techniques during GoCCs (e.g., use vivid imagery, discuss small chance of recovery); response options were presented on a 4-point scale from “Definitely not appropriate” to “Definitely appropriate.”  Specific Attitudes and Behaviors in Goals of Care Conversations during peak-Covid – The survey contained 12 items concerning Covid-specific GoCC attitudes and behaviors. Clinicians reported how often they asked patients with Covid if they wanted a recommendation about LST (e.g., cardiopulmonary resuscitation) decisions (5-point scale ranging from “Never” to “Always”). They also rated the perceived appropriateness of limiting a patient’s LST options (on a 4-point scale from “Definitely not appropriate” to “Definitely appropriate” because of the risk it poses to healthcare providers and because of limited resources for other patients, as well as how concerned they were (on a 5-point scale from “Not at all” to “Extremely”) about resource availability at their facility. Other survey items assessed comfort with prognosticating about whether patients will have outcomes consistent with their goals and values after respiratory failure for both patients with and without Covid (on a 4-point scale from “Very comfortable” to “Very uncomfortable”). Finally, a series of items addressed quality of GoCCs during peak-Covid, including how frequently clinicians felt confident that they provided patients with adequate information to make a fully informed decision (on a 5-point scale from “Never” to “Always”), whether they perceived the quality of their GoCCs to be better or worse compared to pre-pandemic, and the perceived impact of two specific factors on GoCC quality (i.e., restrictions on family/support presence and communicating over telephone). They also reported whether they had at least one GoCC during peak-Covid.  Demographic and Clinical Practice Characteristics – The survey assessed several demographic (i.e., age, gender, race, ethnicity) and clinical practice characteristics [clinical role (i.e., fellow, resident, nurse practitioner or advanced practice nurse, and physician assistant), specialty (i.e., anesthesia, internal medicine, neurology, surgery, pulmonary medicine / critical care, cardiology, geriatrics, palliative care, emergency medicine, and other), year of graduation, percent clinical effort, and percent of clinical effort in inpatient and outpatient settings]. |
| --- | --- | --- | --- | --- |
| Statistical methods | 12 | (*a*) Describe all statistical methods, including those used to control for confounding | 9-10 | We calculated descriptive statistics (counts and proportions) for all demographic and clinical practice characteristics, both for the entire sample (n=323) and for the subsample (n=191) who replied to the open-ended “moral distress” question. We also assessed the frequency and intensity of moral distress. For subsequent analyses, we dichotomized responses to the closed-ended moral distress question into “None”/”Mild”/”Uncomfortable” and “Intense”/”Severe,” with the latter representing heightened moral distress.  We examined associations between demographic and clinical practice characteristics with heightened moral distress during peak Covid-19. We used Chi-square or Kruskal-Wallis tests, as appropriate, to assess zero-order associations of each demographic or clinical practice characteristic, with moral distress. We then conducted multivariable logistic regression to predict moral distress from all demographic and clinical practice characteristics simultaneously, except graduation year and percent clinical effort in inpatient/outpatient settings.  We quantitatively examined bivariate associations between each of the general and specific GoCC attitude and behavior items with moral distress. We dichotomized or trichotomized responses to each general and specific GoCC attitude and behavior item. We used Chi-square or Fisher exact tests, as appropriate, to assess associations with moral distress.  All analyses were conducted using SAS EG 8.3 software. |
|  |  | (*b*) Describe any methods used to examine subgroups and interactions | 9-10 | We calculated descriptive statistics (counts and proportions) for all demographic and clinical practice characteristics, both for the entire sample (n=323) and for the subsample (n=191) who replied to the open-ended “moral distress” question. We also assessed the frequency and intensity of moral distress. For subsequent analyses, we dichotomized responses to the closed-ended moral distress question into “None”/”Mild”/”Uncomfortable” and “Intense”/”Severe,” with the latter representing heightened moral distress.  We examined associations between demographic and clinical practice characteristics with heightened moral distress during peak Covid-19. We used Chi-square or Kruskal-Wallis tests, as appropriate, to assess zero-order associations of each demographic or clinical practice characteristic, with moral distress. We then conducted multivariable logistic regression to predict moral distress from all demographic and clinical practice characteristics simultaneously, except graduation year and percent clinical effort in inpatient/outpatient settings.  We quantitatively examined bivariate associations between each of the general and specific GoCC attitude and behavior items with moral distress. We dichotomized or trichotomized responses to each general and specific GoCC attitude and behavior item. We used Chi-square or Fisher exact tests, as appropriate, to assess associations with moral distress.  All analyses were conducted using SAS EG 8.3 software. |
|  |  | (*c*) Explain how missing data were addressed | 10 | Individuals with missing data on particular survey items were excluded from relevant analyses; we report the number of missing values for any variable within Table footnotes. |
|  |  | (*d*) *Cohort study*—If applicable, explain how loss to follow-up was addressed  *Case-control study*—If applicable, explain how matching of cases and controls was addressed  *Cross-sectional study*—If applicable, describe analytical methods taking account of sampling strategy | N/A | N/A |
|  |  | (*e*) Describe any sensitivity analyses | N/A | N/A |
| Results | | | | |
| Participants | 13* | (a) Report numbers of individuals at each stage of study—eg numbers potentially eligible, examined for eligibility, confirmed eligible, included in the study, completing follow-up, and analysed | 9&11 | p.9, Lines 172-174: We calculated descriptive statistics (counts and proportions) for all demographic and clinical practice characteristics, both for the entire sample (n=323) and for the subsample (n=191) who replied to the open-ended “moral distress” question.  p.11, Lines 215-216: Out of 3,398 eligible clinicians, there were 323 respondents who opted to participate in the survey (response rate 9.5%). |
|  |  | (b) Give reasons for non-participation at each stage | N/A | N/A |
|  |  | (c) Consider use of a flow diagram | N/A | N/A |
| Descriptive data | 14* | (a) Give characteristics of study participants (eg demographic, clinical, social) and information on exposures and potential confounders | 11 | Respondents primarily identified as white (65%) and male (76%), 35% were aged 50-59 years, 58% were attending physicians, and 26% worked in internal medicine or primary care or family medicine. Pre-Covid, respondents spent most of their clinical time in outpatient settings (median 85%; interquartile range: 30-100%) and minimal time in inpatient settings (median 5%; interquartile range: 0-45%). Eighty-one percent of respondents [261/321 (2 respondents did not indicate level of moral distress)] experienced some level of moral distress during peak-Covid [None=19% (60/321), Mild=28% (89/321), Uncomfortable=30% (98/321), Intense=18% (59/321), Severe=5% (15/321)]. See Table 1 for presentation of demographics and clinical practice characteristics by moral distress dichotomized as heightened (“Intense”/“Severe”) or not ( “None”/”Mild”/ ”Uncomfortable”). |
|  |  | (b) Indicate number of participants with missing data for each variable of interest | 12 | Table 1: - 1st footnote: “Two subjects with missing Moral Distress answers were excluded for all analyses”  - Last footnote: “This item [at least one GoCC during the pandemic] had 242 missing values.” |
|  |  | (c) *Cohort study*—Summarise follow-up time (eg, average and total amount) | N/A | N/A |
| Outcome data | 15* | *Cohort study*—Report numbers of outcome events or summary measures over time | N/A | N/A |
|  |  | *Case-control study—*Report numbers in each exposure category, or summary measures of exposure | N/A | N/A |
|  |  | *Cross-sectional study—*Report numbers of outcome events or summary measures | 14 | There were 191 clinicians who provided responses to the open-ended item about circumstances contributing to their peak-Covid moral distress [59% (191/323) of the total survey sample and 73% (191/261) of the subsample indicating any level of moral distress]. The subsample of respondents to the open-ended item was characterized as 68% white, 65% female, 35% aged 50-59 years, 60% worked as an attending physician, and 28% worked in geriatrics or palliative care. Before the pandemic, respondents spent a median of 80% (interquartile range: 30-100%) of their clinical time in the outpatient setting and a median of 10% (interquartile range: 0-50%) of their clinical time in the inpatient setting. (See Table 3.) |
| Main results | 16 | (*a*) Give unadjusted estimates and, if applicable, confounder-adjusted estimates and their precision (eg, 95% confidence interval). Make clear which confounders were adjusted for and why they were included | 12-13 | In bivariate analyses, only gender was significantly associated with experiencing heightened moral distress (Table 1). This effect held in the multivariable logistic regression model (Table 2), with significantly higher reporting of heightened moral distress by women compared to men (OR: 3.35; 95% CI, 1.53-7.37). The multivariable logistic regression also revealed significant differences in moral distress by specialty; compared to those practicing in medical subspecialties other than pulmonary/critical care (e.g., cardiology), the odds of experiencing heightened moral distress were significantly lower in geriatrics/palliative care (OR: 0.40; 95% CI, 0.18-0.87) and internal medicine/family medicine/primary care (OR: 0.46; 95% CI, 0.22-0.98). |
|  |  | (*b*) Report category boundaries when continuous variables were categorized | N/A | N/A |
|  |  | (*c*) If relevant, consider translating estimates of relative risk into absolute risk for a meaningful time period | N/A | N/A |

Continued on next page

| Other analyses | 17 | Report other analyses done—eg analyses of subgroups and interactions, and sensitivity analyses | N/A | N/A |
| --- | --- | --- | --- | --- |
| Discussion | | | | |
| Key results | 18 | Summarise key results with reference to study objectives | 18 | In our mixed methods study, the majority of VA clinicians surveyed experienced at least mild moral distress during early Covid-19 and contributed unique insights into risk factors (i.e., gender and specialty) and contributors (i.e., clinical uncertainty surrounding Covid-19; anticipatory actions; unprecedented restrictions on patient visitation; personal risk to clinicians; and resource shortages) to such moral distress. |
| Limitations | 19 | Discuss limitations of the study, taking into account sources of potential bias or imprecision. Discuss both direction and magnitude of any potential bias | 20 | There are limitations to this study. First, we had a relatively low response rate of 9.5%, though well-distributed variation in respondent characteristics may have minimized the potential for non-response bias. As with all retrospective surveys, there is potential for inaccuracies in recall since respondents reflect on attitudes and behaviors in the past (roughly a year prior in this instance); however, there is no reason to think this would create a bias in one direction or another. Respondents were asked to define peak-Covid based on local factors and thus might not reflect the exact same dates across sites; however, this flexibility allowed us to examine each respondent’s subjective experiences of peak-Covid. Finally, there is the possibility that our findings are not generalizable or transferable to clinicians who work in settings outside of the VA. |
| Interpretation | 20 | Give a cautious overall interpretation of results considering objectives, limitations, multiplicity of analyses, results from similar studies, and other relevant evidence | 18-20 | Gender and specialty were identified as risk factors for moral distress, aligned with findings from other studies. In our study, female clinicians reported higher levels of Covid-related moral distress than male clinicians, similar to results in a Norwegian survey of female hospital-based nurses and physicians. We found clinicians practicing in geriatrics/palliative care and internal medicine/primary care/family medicine were less likely to have heightened moral distress than those in other specialties. There are conflicting findings as to whether clinical specialty is associated with moral harm to clinicians, with a Norwegian study demonstrating cross-specialty differences in moral distress [16] and a U.S. study revealing no differences in moral injury [17]. While comparing results across these studies is a challenge due to variations in study settings and population, it is plausible that the self-selection into geriatrics/palliative care and primary care, combined with the training and philosophy around end-of-life care, may have mitigated some risk of moral distress as found in our study.  New insights from our qualitative themes include two concepts that have not previously been identified as contributors to moral distress in prior literature. This may be because of unique characteristics related to a novel disease (i.e., Theme #1: *Clinical uncertainty surrounding Covid-19*) or because of the need to make decisions prior to the onset of a surge in cases (Theme #2: *Anticipatory actions*). As more information has been gathered over the course of the pandemic, it is possible these factors will not have as great an impact on moral distress in subsequent surges of Covid. Future studies will be needed to both operationalize and validate these concepts as well as to assess their continued association with moral distress.  Meanwhile, one of our qualitative themes is substantiated in extant research (Theme #3: *Unprecedented restrictions on patient visitation*). Visitation restrictions during Covid led to ethical challenges for intensive care nurses in Sweden [18] and emotional distress in intensive care providers globally [19]. Further, visitation restrictions engendered moral distress in Canadian intensive care and medical ward clinicians caring for dying patients [20] and U.S. healthcare workers in outpatient, inpatient, and emergency department settings [17].  Our quantitative and qualitative findings were aligned in identifying the influence of Covid-related *Personal risk to clinicians* (Theme #4) and *Resource shortages* (Theme #5) on clinicians’ moral distress. These two factors are documented as contributing to a sense of anxiety and moral distress or moral injury among a broad array of healthcare disciplines and settings in the U.S. and globally [16, 17, 21, 22].  Ours and others’ findings point to possible leverage points for future intervention. Personal and professional characteristics (gender, specialty) suggest the potential for providing support groups [2, 23] and training and education [17, 20, 23, 24] for clinicians when facing situations at high risk for moral distress. The influences of both clinical uncertainty surrounding Covid and anticipatory actions on moral distress point to the possible benefit of healthcare facilities providing clinicians with not just moral distress consultation services but also ethics consultation services [25]; such a hybrid model would aim to improve healthcare quality by addressing unit-level and system-level contributors to clinician moral distress. Visitation restrictions reinforced the value of video conferencing [17-20] (a communication modality already used in VA for patient care) and also led to a call for clear policies [1, 19] as well as centralized and standardized arbitration of restriction exceptions. Personal risk and resource constraints highlight the need, for example, of transparent guidelines for PPE allocation [1] and, when a clinician is indeed infected, enhanced occupational health options and employment benefits [2, 21]. |
| Generalisability | 21 | Discuss the generalisability (external validity) of the study results | 20 | Finally, there is the possibility that our findings are not generalizable or transferable to clinicians who work in settings outside of the VA. |
| Other information | |  | | |
| Funding | 22 | Give the source of funding and the role of the funders for the present study and, if applicable, for the original study on which the present article is based | 21 | The project was funded by VA Health Services and Research Development Service (PI: Linsky) and supported in part by resources from the VA facilities at which the authors are based. The funder played no role in study design; in data collection, analysis, or interpretation; nor in approving publication of the finalized manuscript. |

*Give information separately for cases and controls in case-control studies and, if applicable, for exposed and unexposed groups in cohort and cross-sectional studies.

**Note:** An Explanation and Elaboration article discusses each checklist item and gives methodological background and published examples of transparent reporting. The STROBE checklist is best used in conjunction with this article (freely available on the Web sites of PLoS Medicine at http://www.plosmedicine.org/, Annals of Internal Medicine at http://www.annals.org/, and Epidemiology at http://www.epidem.com/). Information on the STROBE Initiative is available at www.strobe-statement.org.
