# Supplementary material for: Addressing clinician moral distress: Implications from a mixed methods evaluation during Covid-19
Source: PLoS One. 2023 Sep 15;18(9):e0291542. doi: 10.1371/journal.pone.0291542 (PMC10503769; doi:10.1371/journal.pone.0291542)
Supplement: S1 File — (DOCX) [file pone.0291542.s002.docx]

**S1_File: Survey Instrument**

There is increasing public and professional interest in how healthcare providers (e.g., physicians, nurse practitioners, physician assistants) manage goals of care conversations and life sustaining treatment decisions in the setting of the COVID-19 pandemic. In order to shed light on providers’ perspectives, we are conducting a survey evaluating the impact of COVID-19 on these discussions between patients (or their surrogates) and providers. The survey should take approximately 10 minutes to complete. All responses are confidential.
 
Definitions:
A **life-sustaining treatment** (LST) is a medical treatment that is intended to prolong the life of a patient who would be expected to die soon without the treatment (e.g., artificial nutrition and hydration, mechanical ventilation).
 
A **goals of care conversation** (GoCC) is a conversation between a health care provider and a patient or surrogate for the purpose of determining the patient’s values, goals, and preferences for care, and, based on those factors, making decisions about whether to initiate, limit, or discontinue LSTs.
 
To begin, please think about the time period you would consider as the peak of COVID-19 at your VA Medical Center. If you work at more than one Medical Center, think about the VA facility where you spend most of your time. You can base the peak upon facility caseload, personal workload, increased work hours, increased clinical demand, or something else you feel made it the peak. Please refer to this time point when answering questions about **peak COVID.**Now, please think about a time point in the past **before you were aware of COVID**. Please refer to this time point when answering questions about **pre-COVID**.

Note, this survey will ask you to think about these **two different time periods of peak COVID and pre-COVID**. Please pay careful attention to the section headers and individual questions. **In all cases, please consider conversations with patients and/or their surrogates.**

(End of Page 1 )

First, we would like to learn about you and your clinical practice.

**1.** What is your specialty? (please fill in all that apply)

❑ Anesthesia

❑ Internal Medicine

❑ Neurology

❑ Surgery

❑ Pulmonary Medicine/Critical Care

❑ Cardiology

❑ Geriatrics

❑ Palliative Care

❑ Emergency Medicine

❑ Other ___

**2.** What is your role?

🔾 Attending

🔾 Fellow

🔾 Resident

🔾 Nurse Practitioner or Advanced Practice Nurse (NP or APN)

🔾 Physician Assistant (PA)

**2A.** What year did you graduate from medical school?

🔾 1950

🔾 1951

🔾 1952

🔾 1953

🔾 1954

🔾 1955

🔾 1956

🔾 1957

🔾 1958

🔾 1959

🔾 1960

🔾 1961

🔾 1962

🔾 1963

🔾 1964

🔾 1965

🔾 1966

🔾 1967

🔾 1968

🔾 1969

🔾 1970

🔾 1971

🔾 1972

🔾 1973

🔾 1974

🔾 1975

🔾 1976

🔾 1977

🔾 1978

🔾 1979

🔾 1980

🔾 1981

🔾 1982

🔾 1983

🔾 1984

🔾 1985

🔾 1986

🔾 1987

🔾 1988

🔾 1989

🔾 1990

🔾 1991

🔾 1992

🔾 1993

🔾 1994

🔾 1995

🔾 1996

🔾 1997

🔾 1998

🔾 1999

🔾 2000

🔾 2001

🔾 2002

🔾 2003

🔾 2004

🔾 2005

🔾 2006

🔾 2007

🔾 2008

🔾 2009

🔾 2010

🔾 2011

🔾 2012

🔾 2013

🔾 2014

🔾 2015

🔾 2016

🔾 2017

🔾 2018

🔾 2019

🔾 2020

🔾 2021

🔾 2022

🔾 2023

🔾 2024

**2A.** What year did you graduate with your advanced degree?

🔾 1950

🔾 1951

🔾 1952

🔾 1953

🔾 1954

🔾 1955

🔾 1956

🔾 1957

🔾 1958

🔾 1959

🔾 1960

🔾 1961

🔾 1962

🔾 1963

🔾 1964

🔾 1965

🔾 1966

🔾 1967

🔾 1968

🔾 1969

🔾 1970

🔾 1971

🔾 1972

🔾 1973

🔾 1974

🔾 1975

🔾 1976

🔾 1977

🔾 1978

🔾 1979

🔾 1980

🔾 1981

🔾 1982

🔾 1983

🔾 1984

🔾 1985

🔾 1986

🔾 1987

🔾 1988

🔾 1989

🔾 1990

🔾 1991

🔾 1992

🔾 1993

🔾 1994

🔾 1995

🔾 1996

🔾 1997

🔾 1998

🔾 1999

🔾 2000

🔾 2001

🔾 2002

🔾 2003

🔾 2004

🔾 2005

🔾 2006

🔾 2007

🔾 2008

🔾 2009

🔾 2010

🔾 2011

🔾 2012

🔾 2013

🔾 2014

🔾 2015

🔾 2016

🔾 2017

🔾 2018

🔾 2019

🔾 2020

🔾 2021

🔾 2022

🔾 2023

🔾 2024

**2A.** What year did you graduate from Physician Assistant school?

🔾 1950

🔾 1951

🔾 1952

🔾 1953

🔾 1954

🔾 1955

🔾 1956

🔾 1957

🔾 1958

🔾 1959

🔾 1960

🔾 1961

🔾 1962

🔾 1963

🔾 1964

🔾 1965

🔾 1966

🔾 1967

🔾 1968

🔾 1969

🔾 1970

🔾 1971

🔾 1972

🔾 1973

🔾 1974

🔾 1975

🔾 1976

🔾 1977

🔾 1978

🔾 1979

🔾 1980

🔾 1981

🔾 1982

🔾 1983

🔾 1984

🔾 1985

🔾 1986

🔾 1987

🔾 1988

🔾 1989

🔾 1990

🔾 1991

🔾 1992

🔾 1993

🔾 1994

🔾 1995

🔾 1996

🔾 1997

🔾 1998

🔾 1999

🔾 2000

🔾 2001

🔾 2002

🔾 2003

🔾 2004

🔾 2005

🔾 2006

🔾 2007

🔾 2008

🔾 2009

🔾 2010

🔾 2011

🔾 2012

🔾 2013

🔾 2014

🔾 2015

🔾 2016

🔾 2017

🔾 2018

🔾 2019

🔾 2020

🔾 2021

🔾 2022

🔾 2023

🔾 2024

**3.** Do you consider yourself Hispanic or of Spanish or Latin-American origin?

🔾 Yes

🔾 No

🔾 Prefer not to answer

**4.** How do you classify your race? (please check all that apply)

❑ American Indian or Alaskan Native

❑ Asian

❑ Black or African American

❑ White or Caucasian 

❑ Other ___

❑ Prefer not to answer

**5.** What is your gender?

🔾 Female

🔾 Male

🔾 Non-binary

🔾 Prefer not to answer

**6.** What is your age?

🔾 20-29

🔾 30-39

🔾 40-49

🔾 50-59

🔾 60-69

🔾 70+

**7. Pre-COVID**, what percentage of your time at work was devoted to clinical duties?

🔾 None

🔾 >0%-20%

🔾 21%-40%

🔾 41%-60%

🔾 61%-80%

🔾 81%-100%

**8. Pre-COVID,** what percentage of your clinical time was devoted to the following settings? Answers should add to 100%.

Inpatient                                          ________________________________________

Outpatient                                        ________________________________________

Community Living Center                ________________________________________

**9.** Did your percentage of time for clinical duties change during **peak COVID**?

🔾 Yes, it increased in my usual clinical setting

🔾 Yes, it increased in a different clinical setting

🔾 No, it stayed the same

🔾 Yes, it decreased

(End of Page 2 )

Next, we would like to understand your experience with goals of care conversations during peak COVID. Please answer these questions thinking about **all** patients during **peak COVID**.

**10.** In a typical week, how many goals of care conversations did you have with patients?

🔾 None

🔾 1-2

🔾 3-4

🔾 5 or more

**11.** How does this compare to pre-COVID?

🔾 I had more goals of care conversations during peak COVID

🔾 I had about the same number of goals of care conversations during peak COVID

🔾 I had fewer goals of care conversations during peak COVID

**12.** How often did you document your GoCCs...

|  | Never | Rarely | Sometimes | Often | Always | N/A - I had no GoCCs |
| --- | --- | --- | --- | --- | --- | --- |
| Using VA’s “Life-Sustaining Treatment” template note? | 🔾 | 🔾 | 🔾 | 🔾 | 🔾 | 🔾 |
| As free text within your progress note? | 🔾 | 🔾 | 🔾 | 🔾 | 🔾 | 🔾 |

(End of Page 3 )

We would like to know more about your thoughts on providing patients with recommendations for life sustaining treatment decisions pre-COVID. Please answer these questions thinking about **all** patients **pre-COVID**.

**13.** In general, how appropriate or inappropriate was it for healthcare providers (e.g., physicians, physician assistants, nurse practitioners) to provide patients with specific recommendations about life sustaining treatment decisions?

🔾 Very appropriate

🔾 Somewhat appropriate

🔾 Somewhat inappropriate

🔾 Very inappropriate

**14.** In general, how comfortable or uncomfortable were you with making specific recommendations to patients about life sustaining treatment decisions?

🔾 Very comfortable

🔾 Somewhat comfortable

🔾 Somewhat uncomfortable

🔾 Very uncomfortable

**15.** How often, if ever, did you ask patients if they wanted your recommendations about life sustaining treatment decisions?

🔾 Never

🔾 Rarely

🔾 Sometimes

🔾 Often

🔾 Always

🔾 Not applicable - I did not have any goals of care conversations

**16.** Please indicate whether you agree or disagree with the following statements:

**Providing a patient with a specific recommendation about life sustaining treatment:**

|  | Disagree strongly | Disagree somewhat | Agree somewhat | Agree strongly |
| --- | --- | --- | --- | --- |
| Is a healthcare provider’s duty | 🔾 | 🔾 | 🔾 | 🔾 |
| Is appropriate only if the patient wants the recommendation | 🔾 | 🔾 | 🔾 | 🔾 |
| Makes it easier for the patient to make life sustaining treatment decisions | 🔾 | 🔾 | 🔾 | 🔾 |
| Further burdens the patient | 🔾 | 🔾 | 🔾 | 🔾 |
| Unduly influences the patient’s decision | 🔾 | 🔾 | 🔾 | 🔾 |
| Places too great a burden on the provider | 🔾 | 🔾 | 🔾 | 🔾 |

**17.** Consider those situations in which a provider favored limiting (i.e., not initiating) a patient’s life sustaining treatment. In such cases, how **ethically appropriate** would it have been for a provider to:

|  | Definitely not appropriate | Probably not appropriate | Probably appropriate | Definitely appropriate |
| --- | --- | --- | --- | --- |
| Discourage interventions by using vivid imagery (e.g., “CPR would break your ribs…”) | 🔾 | 🔾 | 🔾 | 🔾 |
| Recommend a time-limited trial of therapy | 🔾 | 🔾 | 🔾 | 🔾 |
| Focus the discussion on the medical facts that support the choice the provider thinks is best for the patient | 🔾 | 🔾 | 🔾 | 🔾 |
| Divide the decision about limiting life support into smaller decisions about individual interventions | 🔾 | 🔾 | 🔾 | 🔾 |
| Discuss the patient’s small chance of recovery | 🔾 | 🔾 | 🔾 | 🔾 |
| Explain what the provider would do if the patient were the provider’s family member | 🔾 | 🔾 | 🔾 | 🔾 |
| Emphasize those patient values that support the choice the provider thinks is best for the patient | 🔾 | 🔾 | 🔾 | 🔾 |
| Discuss the provider’s prognostic uncertainty with the patient or family | 🔾 | 🔾 | 🔾 | 🔾 |
| Make an independent treatment decision and inform the patient of that decision | 🔾 | 🔾 | 🔾 | 🔾 |

(End of Page 4 )

Now we will **change time periods** and consider peak COVID. We would like to understand your thoughts on providing recommendations for life sustaining treatment decisions during peak COVID. Please answer these next questions thinking about **patients with suspected or confirmed COVID** during **peak COVID**.

**18.** In general, how appropriate or inappropriate is it for healthcare providers to provide COVID patients with specific recommendations about life sustaining treatment decisions?

🔾 Very appropriate

🔾 Somewhat appropriate

🔾 Somewhat inappropriate

🔾 Very inappropriate

**19.** In general, how comfortable or uncomfortable are you with making specific recommendations to COVID patients about life sustaining treatment decisions?

🔾 Very comfortable

🔾 Somewhat comfortable

🔾 Somewhat uncomfortable

🔾 Very uncomfortable

**20.** How often, if ever, do you ask COVID patients if they want your recommendations about life sustaining treatment decisions?

🔾 Never

🔾 Rarely

🔾 Sometimes

🔾 Often

🔾 Always

🔾 Not applicable - I haven't directly cared for COVID patients

**21.** Please indicate whether you agree or disagree with the following statements:

**Providing a COVID patient with a specific recommendation about life sustaining treatment:**

|  | Disagree strongly | Disagree somewhat | Agree somewhat | Agree strongly |
| --- | --- | --- | --- | --- |
| Is a healthcare provider’s duty | 🔾 | 🔾 | 🔾 | 🔾 |
| Is appropriate only if the patient wants the recommendation | 🔾 | 🔾 | 🔾 | 🔾 |
| Makes it easier for the patient to make life sustaining treatment decisions | 🔾 | 🔾 | 🔾 | 🔾 |
| Further burdens the patient | 🔾 | 🔾 | 🔾 | 🔾 |
| Unduly influences the patient’s decision | 🔾 | 🔾 | 🔾 | 🔾 |
| Places too great a burden on the provider | 🔾 | 🔾 | 🔾 | 🔾 |

**22.** Consider those situations in which a provider favors limiting (i.e., not initiating) a COVID patient’s life sustaining treatment. In such cases, how **ethically appropriate** is it for a provider to:

|  | Definitely not appropriate | Probably not appropriate | Probably appropriate | Definitely appropriate |
| --- | --- | --- | --- | --- |
| Discourage interventions by using vivid imagery (e.g., “CPR would break your ribs…”) | 🔾 | 🔾 | 🔾 | 🔾 |
| Recommend a time-limited trial of therapy | 🔾 | 🔾 | 🔾 | 🔾 |
| Focus the discussion on the medical facts that support the choice the provider thinks is best for the patient | 🔾 | 🔾 | 🔾 | 🔾 |
| Divide the decision about limiting life support into smaller decisions about individual interventions | 🔾 | 🔾 | 🔾 | 🔾 |
| Discuss the patient’s small chance of recovery | 🔾 | 🔾 | 🔾 | 🔾 |
| Explain what the provider would do if the patient were the provider’s family member | 🔾 | 🔾 | 🔾 | 🔾 |
| Emphasize those patient values that support the choice the provider thinks is best for the patient | 🔾 | 🔾 | 🔾 | 🔾 |
| Discuss the provider’s prognostic uncertainty with the patient or family | 🔾 | 🔾 | 🔾 | 🔾 |
| Make an independent treatment decision and inform the patient of that decision | 🔾 | 🔾 | 🔾 | 🔾 |

**23.** In general, how appropriate or inappropriate is it for providers to limit a COVID patient’s decision regarding life sustaining treatment because of each of the following:

|  | Definitely not appropriate | Probably not appropriate | Probably appropriate | Definitely appropriate |
| --- | --- | --- | --- | --- |
| The risk it poses on providers (e.g., inadequate personal protective equipment) | 🔾 | 🔾 | 🔾 | 🔾 |
| Limited resources for other patients (e.g.,ventilators, beds) | 🔾 | 🔾 | 🔾 | 🔾 |

(End of Page 5 )

We would now like to learn more about your thoughts about goals of care conversations and life sustaining treatment decision making that you personally experienced during peak COVID. Unless directed otherwise, please answer these next questions thinking about **all** patients during **peak COVID**.

**24.** How comfortable or uncomfortable are you with prognosticating about whether a patient will have an outcome consistent with their goals and values after respiratory failure?

|  | Very comfortable | Somewhat comfortable | Somewhat uncomfortable | Very uncomfortable |
| --- | --- | --- | --- | --- |
| For patients in general, without COVID | 🔾 | 🔾 | 🔾 | 🔾 |
| For patients with suspected or confirmed COVID | 🔾 | 🔾 | 🔾 | 🔾 |

**25.** Did you have at least one discussion with a patient about life sustaining treatment decisions during peak COVID?

🔾 Yes

🔾 No

(End of Page 6 )

**26.** Please think about your discussions with all patients about life sustaining treatment decisions (e.g., CPR, mechanical ventilation, renal replacement therapy). How often, if at all, do you do the following?

|  | Never | Rarely | Sometimes | Often | Always |
| --- | --- | --- | --- | --- | --- |
| Identify the patient’s healthcare proxy or surrogate | 🔾 | 🔾 | 🔾 | 🔾 | 🔾 |
| Provide medical information to the patient | 🔾 | 🔾 | 🔾 | 🔾 | 🔾 |
| Engage in deliberations with the patient about the decision | 🔾 | 🔾 | 🔾 | 🔾 | 🔾 |
| Make a specific recommendation about life sustaining treatment decisions (e.g., choose Do Not Resuscitate vs. Full Code) | 🔾 | 🔾 | 🔾 | 🔾 | 🔾 |
| Encourage the patient to articulate their values | 🔾 | 🔾 | 🔾 | 🔾 | 🔾 |
| Suggest that a particular decision is most consistent with the patient’s values | 🔾 | 🔾 | 🔾 | 🔾 | 🔾 |
| Make an independent treatment decision and inform the patient of that decision | 🔾 | 🔾 | 🔾 | 🔾 | 🔾 |
| Tell the patient that decisions regarding code status can be changed at any time | 🔾 | 🔾 | 🔾 | 🔾 | 🔾 |

**27.** After you have a goals of care conversation with a patient, how often do you feel confident that you provided the patient with adequate information to make a fully informed decision?

🔾 Never

🔾 Rarely

🔾 Sometimes

🔾 Often

🔾 Always

**28.** In general, how would you compare the quality of your goals of care conversations during **peak COVID** to those during **pre-COVID**?

🔾 Much better during peak COVID

🔾 Somewhat better during peak COVID

🔾 About the same during peak COVID

🔾 Somewhat worse during peak COVID

🔾 Much worse during peak COVID

🔾 Not applicable - I did not have GoCCs during both pre-COVID and peak COVID

**29.** Some of the following situations may have occurred during peak COVID. How do you think each of these situations affected the quality of goals of care conversations?

Made the quality of GoCCs...

|  | Much better | Better | About the same | Worse | Much worse | Did not experience |
| --- | --- | --- | --- | --- | --- | --- |
| Restrictions on family/support system presence during the conversation | 🔾 | 🔾 | 🔾 | 🔾 | 🔾 | 🔾 |
| Communicating over the telephone (compared to in-person) | 🔾 | 🔾 | 🔾 | 🔾 | 🔾 | 🔾 |
| Communicating over video (compared to in-person) | 🔾 | 🔾 | 🔾 | 🔾 | 🔾 | 🔾 |

**30.** How concerned were you about resource availability at your facility (e.g., personal protective equipment, beds, ventilators, staff)?

🔾 Not at all

🔾 Slightly

🔾 Moderately

🔾 Very

🔾 Extremely

(End of Page 7 )

Finally, we would like to ask you about moral distress. Moral distress occurs when professionals are unable to carry out what they believe to be ethically appropriate actions because of internal (e.g. fear of repercussions; self-doubt) or external (e.g. lack of support, hierarchies of healthcare system) constraints.

**31.** Please rate the intensity of your overall level of moral distress during **peak COVID**.

🔾 None

🔾 Mild

🔾 Uncomfortable

🔾 Intense

🔾 Severe

**31A.** Can you tell us more about the circumstances that may have contributed to these feelings?

______________________________________________________________

______________________________________________________________

______________________________________________________________

**32.** Is there anything else you would like to add about how COVID influenced your goals of care conversations and life sustaining treatment decision making with patients?

______________________________________________________________

______________________________________________________________

______________________________________________________________

**33.** Would you be willing to participate in a brief telephone interview to discuss your experiences with goals of care conversations during the COVID pandemic?

🔾 Yes

🔾 No

(End of Page 8 )
