# Supplementary material for: Addressing clinician moral distress: Implications from a mixed methods evaluation during Covid-19
Source: PLoS One. 2023 Sep 15;18(9):e0291542. doi: 10.1371/journal.pone.0291542 (PMC10503769; doi:10.1371/journal.pone.0291542)
Supplement: S1 Table — (DOCX) [file pone.0291542.s003.docx]

| S1 Table: Original Sources of Individual Survey Items | | | |
| --- | --- | --- | --- |
| Survey Item | Developed by Authors | Developed by Binder et al., 2016 | Developed by Brush et al., 2012 |
| 1. What is your specialty? (please fill in all that apply) |  |  | X* |
| 2. What is your role | X |  |  |
| This Question is Conditionally Shown if: (2 = Attending OR2 = Fellow OR2 = Resident)  What year did you graduate from medical school? | X |  |  |
| This Question is Conditionally Shown if: (2 = Nurse Practitioner or Advanced Practice Nurse  (NP or APN))  2A. What year did you graduate with your advanced degree? | X |  |  |
| This Question is Conditionally Shown if: (2 = Physician Assistant (PA))  2A. What year did you graduate from Physician Assistant school? | X |  |  |
| 3. Do you consider yourself Hispanic or of Spanish or Latin-American origin? |  |  | X* |
| 4. How do you classify your race? |  |  | X* |
| 5. What is your gender? | X |  |  |
| 6. What is your age? | X |  |  |
| 7. Pre-COVID, what percentage of your time at work was devoted to clinical duties? | X |  |  |
| This Question is Conditionally Hidden if: (7 = None )  8. Pre-COVID, what percentage of your clinical time was devoted to the following settings:  Inpatient  Outpatient  Community Living Center  (answers should add to 100)? | X |  |  |
| 9. Did your percentage of time for clinical duties change during peak COVID? | X |  |  |
| 10. In a typical week, how many goals of care conversations did you have with patients? |  |  | X† |
| 11. How does this compare to pre-COVID? | X |  |  |
| This Question is Conditionally Hidden if: (7 = None AND9 = No, it stayed the same)  12. How often did you document your GoCCs in the electronic medical record..  …using VA’s “Life-Sustaining Treatment” template note  … as free text within your progress note? | X |  |  |
| 13. In general, how appropriate or inappropriate was it for healthcare providers (e.g.,  physicians, physician assistants, nurse practitioners) to provide patients with specific  recommendations about life sustaining treatment decisions? |  |  | X† |
| 14. In general, how comfortable or uncomfortable were you with making specific  recommendations to patients about life sustaining treatment decisions? |  |  | X† |
| 15. How often, if ever, did you ask patients if they wanted your recommendations about life sustaining treatment decisions? |  |  | X† |
| 16. Please indicate whether you agree or disagree with the following statements:  Providing a patient with a specific recommendation about life sustaining treatment…  a. Is a healthcare  provider’s duty  b. Is appropriate only if the patient wants the recommendation  c. Makes it easier for the patient to make life sustaining treatment decisions  d. Further burdens the patient  e. Unduly influences the patient’s decision  f. Places too great a burden on the provider |  |  | X† |
| 17. Consider those situations in which a provider favored limiting (i.e., not initiating) a patient’s life sustaining treatment. In such cases, how ethically appropriate would it have been for a provider to:  a. Discourage interventions by  using vivid imagery (e.g.,  “CPR would break your  ribs…”)  b. Recommend a time-limited trial of therapy  c. Focus the discussion on the  medical facts that support the  choice the provider thinks is  best for the patient  d. Divide the decision about  limiting life support into  smaller decisions about individual interventions  e. Discuss the patient’s small  chance of recovery  f. Explain what the provider would do if the patient  were the provider’s family  member  g. Emphasize those  patient values  that support the  choice the provider thinks is  best for the patient  h. Discuss the provider’s  prognostic uncertainty with  the patient or family  i. Make an independent  treatment decision and  inform the patient of that decision |  |  | X† |
| 18. In general, how appropriate or inappropriate is it for healthcare providers to provide  COVID patients with specific recommendations about life sustaining treatment decisions? |  |  | X† |
| 19. In general, how comfortable or uncomfortable are you with making specific  recommendations to COVID patients about life sustaining treatment decisions? |  |  | X† |
| 20. How often, if ever, do you ask COVID patients if they want your recommendations about  life sustaining treatment decisions? |  |  | X† |
| 21. Please indicate whether you agree or disagree with the following statements:  Providing a COVID patient with a specific recommendation about life sustaining treatment:  a. Is a healthcare provider’s duty  b. Is appropriate only if the patient wants the  recommendation  c. Makes it easier for the patient to make life sustaining treatment decisions  d. Further burdens the patient  e. Unduly influences the patient’s decision  f. Places too great a  burden on the provider |  |  | X† |
| 22. Consider those situations in which a provider favored limiting (i.e., not initiating) a patient’s life sustaining treatment. In such cases, how ethically appropriate would it have been for a provider to:  a. Discourage interventions by  using vivid imagery (e.g.,  “CPR would break your  ribs…”)  b. Recommend a time-limited trial of therapy  c. Focus the discussion on the  medical facts that support the  choice the provider thinks is  best for the patient  d. Divide the decision about  limiting life support into  smaller decisions about individual interventions  e. Discuss the patient’s small  chance of recovery  f. Explain what the provider would do if the patient  were the provider’s family  member  g. Emphasize those  patient values  that support the  choice the provider thinks is  best for the patient  h. Discuss the provider’s  prognostic uncertainty with  the patient or family  i. Make an independent  treatment decision and  inform the patient of that decision |  |  | X† |
| 23. In general, how appropriate or inappropriate is it for providers to limit a COVID  patient’s decision regarding life sustaining treatment because of each of the following:  a. the risk it poses on providers (e.g., , inadequate PPE).  b. limited resources for other patients (e.g., ventilators, beds). |  |  | X† |
| 24. How comfortable or uncomfortable are you with prognosticating about whether a patient will have an outcome consistent with their goals and values after respiratory failure?^x^  a. For patients in  general, without  COVID  b. For patients  with suspected  or confirmed  COVID |  |  | X† |
| This Question is Conditionally Hidden if: (10 ≠ None OR(7 = None AND9 = No, it stayed the  same))  25. Did you have at least one discussion with a patient about life sustaining treatment decisions during peak COVID? |  |  | X† |
| 26. Please think about your discussions with all patients about life sustaining treatment  decisions (e.g., CPR, mechanical ventilation, renal replacement therapy). How often, if  at all, do you do the following?  a. Identify the  patient’s healthcare proxy  or surrogate  b. Provide medical information to the patient  c. Engage in deliberations with  the patient about the decision  d. Make a specific recommendation about life  sustaining treatment decisions (e.g., choose Do Not Resuscitate vs. Full Code)  e. Encourage the patient to  articulate their values  f. Suggest that a particular  decision is most consistent with  the patient’s values  g. Make an independent  treatment decision and  inform the patient of that decision  h. Tell the patient that decisions  regarding code status can be  changed at any time |  | X† | X† |
| This Question is Conditionally Hidden if: (25 = No OR(7 = None AND9 = No, it stayed the  same))  27. After you have a goals of care conversation with a patient, how often do you feel confident  that you provided the patient with adequate information to make a fully informed decision? |  | X† |  |
| This Question is Conditionally Hidden if: (25 = No OR(7 = None AND9 = No, it stayed the  same))  28. In general, how would you compare the quality of your goals of care conversations during  peak COVID to those during pre-COVID? | X |  |  |
| This Question is Conditionally Hidden if: (25 = No OR(7 = None AND9 = No, it stayed the  same))  29. Some of the following situations may have occurred during peak COVID. How do  you think each of these situations affected the quality of goals of care conversations?  Restrictions on family/support  system presence during the  conversation  Communicating over the  telephone or video  (compared to  in-person) | X |  |  |
| 30. How concerned were you about resource availability at your facility (e.g., personal protective  equipment, beds, ventilators, staff)? | X |  |  |
| 31. Please rate the intensity of your overall level of moral distress during peak COVID.  This Question is Conditionally Shown if: (31 = Mild OR31 = Uncomfortable OR31 = Intense  OR31 = Severe)  31A. Can you tell us more about the circumstances that may have contributed to these  feelings? | X |  |  |
| 32. Is there anything else you would like to add about how COVID influenced your goals of  care conversations and life sustaining treatment decision making with patients? | X |  |  |
| 33. Would you be willing to participate in a brief telephone interview to discuss your  experiences with goals of care conversations during the COVID pandemic? | X |  |  |

* Survey item was used verbatim from source

† Survey item was modified for our purposes

Binder AF, Huang GC, Buss MK. Uninformed consent: do medicine residents lack the proper framework for code status discussions? J Hosp Med. 2016;11(2):111-6. [PMID: 26471452] doi: 10.1002/jhm.2497

Brush DR, Rasinski KA, Hall JB, Alexander GC. Recommendations to limit life support. American Journal of Respiratory and Critical Care Medicine. 2012;186(7):633-9. doi: 10.1164/rccm.201202-0354OC.
