# Supplementary material for: Addressing clinician moral distress: Implications from a mixed methods evaluation during Covid-19
Source: PLoS One. 2023 Sep 15;18(9):e0291542. doi: 10.1371/journal.pone.0291542 (PMC10503769; doi:10.1371/journal.pone.0291542)
Supplement: S2 Table — (DOCX) [file pone.0291542.s004.docx]

| S2 Table: Codebook for Qualitative Analysis | |
| --- | --- |
| Codes | Code Definitions |
| Agency | Internal - Personal expectations or feelings about one's own performance, efficacy, and ability to help patients/family as would like to |
| Allocation decisions | Influence on moral distress/distress of - or opinions about - need to ration care (or not) |
| Anticipatory anxiety | Policies or behaviors pre-empting the need to triage resources |
| Changing job parameters | Influence of changes in work conditions (e.g., work hours, setting of care) on presence of moral distress/distress |
| Community resources | Influence of in/sufficient community resources on moral distress/distress |
| Covid-19 | The unique circumstances that Covid-19 has created which influence moral distress/distress |
| General distress | When distress seems not to be based on internal/external constraints which lead to violation of the provider's values or sense of ethics but more based on heightened levels of stress |
| EoL discussion | Reference to an end-of-life discussion held between the provider and a patient/family |
| External influences on LST decisions | Structural, procedural, or other persons (outside of the patient-provider dyad) influencing LST decisions being made by the patient or provider |
| HCP risk - personal/family/colleagues | Includes presence or absence of infection risk to self or family or colleagues; refers to risk of spread plus risk due to having vulnerable conditions (older age and medical conditions) and/or potential influence on desire to fulfil professional obligations |
| In/sufficiency of information | Presence or absence of timely or adequate levels of information |
| Influencing LST decision | Specifically recounting instances where responding HCP felt they or other staff did this |
| Isolation | Influence of patient isolation from family or from providers (due to e.g., visitation policies, etc.) on moral distress/distress |
| Judgment | Includes providers assigning responsibility to patient for acquiring COVID; perceptions of patients as demanding |
| Leadership support | Presence or absence of support from supervisors or organizational leaders |
| Legal | Anything related to concerns of litigation or lawsuits |
| Limited moral distress | Participant directly states that they are not experiencing moral disequilibrium, that moral distress has been mitigated by other factors, or that their moral distress is not significant |
| Modality of care | Influence of using telehealth or face to face, etc. on moral distress/distress |
| Non-Covid-19 care | Impact on care for patients without Covid-19, delay of usual care due to fear of Covid-19 exposure |
| Not knowing the patient | Influence of how provider or family knowing / not knowing the patient or his/her wishes sufficiently on moral distress/distress |
| Obligation | External pressure to fulfill professional requirements |
| Overwhelmed | Verbatim use of the word "overwhelmed" or any derivative of this word or any close synonym |
| Patient characteristics | Patient/family demographics or patient diagnosis / medical condition that influence presence of moral distress / distress |
| Patient/family distress | Includes presence or absence of patient and family distress including fear and anxiety |
| “Playing God” | Provider being in the situation of deciding who lives or dies |
| Policy/plan/protocol | Implications of VA- or facility-level policies and plans and protocols and their impact on moral distress/distress |
| Politics | Influence of national politics, politicization of issues |
| Professional experience | Influence of past professional experience (e.g., experience or not with other challenging circumstances) on presence of moral distress/distress |
| Prognostication | Challenges or lack thereof in the ability to gauge a patient's prognosis and resulting influence on moral distress/distress |
| Quality of care | Influence of quantity (duration of care episode, whether any care episode) and quality or lack thereof of episode of care (e.g. superficial interaction, ability to follow preferred practices) on moral distress/stress |
| Resource availability | Availability or lack thereof of resources (e.g., supplies, personnel, time, money) |
| Risk of [being a] vector | Provider concerns of their being a vector of infection spread, not wanting to cause harm |
| Setting of care | The circumstances unique to the setting of care (ICU, primary care, etc.) which influence moral distress/stress |
| Spiritual support | Presence or absence of chaplains and other spiritual leaders |
| Team dynamics | Absence or presence at the team level of communication, collaboration or teamwork/support, positive relationship(s) - (teams being broadly defined) |
| Uncertainty | A code to capture any broad mention of uncertainty, e.g., "the stress of the unknown" |
| Uncertainty – Clinical | Influence of provider feeling un/certain about how to proceed with clinical care on moral distress/distress |
| Under/valued skills | Provider feeling that VA leadership in or out of the specific facility or supervisors or fellow team members or general population do not adequately appreciate the provider's skill set |
| Volume of death | Impact of number of deaths on presence of moral distress/distress |
| Workload | Influence of demands of caseload and/or other job requirements on presence of moral distress/distress or on provider outcomes (like physical exhaustion, fatigue) |
