# Supplementary material for: Addressing clinician moral distress: Implications from a mixed methods evaluation during Covid-19
Source: PLoS One. 2023 Sep 15;18(9):e0291542. doi: 10.1371/journal.pone.0291542 (PMC10503769; doi:10.1371/journal.pone.0291542)
Supplement: S3 Table — (DOCX) [file pone.0291542.s005.docx]

| S3 Table:  Demographic Information of Study Sample vs. Total Eligible Sample | | |
| --- | --- | --- |
|  | Study Sample  (N=321^*^) | Total Eligible Sample (N=3,396) |
| **Age**, n (%) |  |  |
| 20-39 | 42 (13) | 348 (10) |
| 40-49 | 83 (26) | 739 (22) |
| 50-59 | 109 (34) | 875 (26) |
| 60+ | 87 (27) | 968 (29) |
| Missing | 0(0) | 466 (14) |
|  |  |  |
| **Gender**, n (%) |  |  |
| Male | 112 (35) | 1087 (32) |
| Female | 201 (63) | 1477 (43) |
| Missing | 0(0) | 832 (24) |
|  |  |  |
| **Role**, n (%) |  |  |
| Physician | 186 (58) | 2158 (64) |
| Advanced Nurse Practitioner/ Physician Assistant | 135 (42) | 1238 (36) |
|  |  |  |
| **Specialty**, n (%) |  |  |
| Internal Medicine/Primary Care/  Family Medicine | 89 (28) | 1523 (45^†^) |
| Geriatrics/Palliative Care | 85 (26) | 291 (9^†^) |
| Emergency Medicine/Pulmonary Medicine/  Critical Care | 48 (15) | 472 (14^†^) |
| Other^‡^ | 99 (31) | 1110 (33^†^) |
| ^*^Two subjects with missing Moral Distress answers were excluded for all analyses; ^†^ Total percentage exceeds 100% due to rounding error; ^‡^Reported “Other” specialties include anticoagulation services, allergy and immunology, addiction medicine, cardiology, dermatology, endocrinology, employee health services, hematology/oncology, infectious disease, nephrology, neurology/traumatic brain injury, occupational health services, psychiatry/mental health, pain medicine, rheumatology, radiologic services, surgery, wound care, and unspecified. | | |
