# Supplementary material for: Addressing clinician moral distress: Implications from a mixed methods evaluation during Covid-19
Source: PLoS One. 2023 Sep 15;18(9):e0291542. doi: 10.1371/journal.pone.0291542 (PMC10503769; doi:10.1371/journal.pone.0291542)
Supplement: S4 Table — (RTF) [file pone.0291542.s006.rtf]

S4 Table: Respondent Characteristics (Total and Comparative by Each Level of Moral Distress)
	
									
	Total
(N=321*)
n (%)	None
(N=60)
n (%)	Mild
(N=89)
n (%)	Uncomfortable
(N=98)
n (%)	Intense
(N=59)
n (%)	Severe
(N=15)
n (%)	

P-value		
Age							0.61†		
20-39	42 (13)	7 (12)	10 (11)	14 (14)	9 (15)	2 (13)			
40-49	83 (26)	13 (22)	24 (27)	23 (23)	19 (32)	4 (27)			
50-59	109 (34)	18 (30)	27 (30)	40 (41)	20 (34)	4 (27)			
60+	87 (27)	22 (37)	28 (31)	21 (21)	11 (19)	5 (33)			
Gender							0.021		
Male	112 (35)	25 (42)	40 (45)	31 (32)	14 (24)	2 (13)			
Female	201 (63)	35 (58)	45 (51)	64 (65)	45 (76)	12 (80)			
Race							0.28†		
White	207 (64)	37 (62)	65 (73)	63 (64)	34 (58)	8 (53)			
Non-White	114 (36)	23 (38)	24 (27)	35 (36)	25 (42)	7 (47)			
Ethnicity							0.27†		
Hispanic	18 (6)	1 (2)	3 (3)	9 (9)	4 (7)	1 (7)			
Non-Hispanic	303 (94)	59 (98)	86 (97)	89 (91)	55 (93)	14 (93)			
Role							0.31†		
Physician	186 (58)	34 (57)	60 (67)	53 (54)	31 (53)	8 (53)			
Advanced Nurse Practitioner/    
 Physician Assistant	135 (42)	26 (43)	29 (33)	45 (46)	28 (47)	7 (47)			
Specialty							0.45†		
Internal Medicine/Primary Care/     
 Family Medicine	89 (28)	20 (33)	30 (34)	24 (24)	14 (24)	1 (7)			
Geriatrics/Palliative Care	85 (26)	17 (28)	19 (21)	32 (33)	13 (22)	4 (27)			
Emergency Medicine/Pulmonary   Medicine/Critical Care	48 (15)	6 (10)	13 (15)	15 (15)	10 (17)	4 (27)			
Other§	99 (31)	17 (28)	27 (30)	27 (28)	22 (37)	6 (40)			
Year of Graduation, Median (IQR)							0.20‡		
	2000 (1989-2010)	1999 (1987-2009)	1999 (1987-2008)	1999 (1993-2011)	2003 (1993-2011)	2000 (1986-2011)			
Clinical Effort							0.45†		
0-80%	147 (46)	27 (45)	47 (53)	45 (46)	22 (37)	6 (40)			
81%-100%	174 (54)	33 (55)	42 (47)	53 (54)	37 (63)	9 (60)			
Clinical Setting, Median (IQR)							0.48‡		
Inpatient	5 (0-45)	0 (0-27)	5 (0-40)	3 (0-50)	13 (0-50)	0 (0-35)			
Outpatient	85 (30-100)	88 (10-100)	90 (40-100)	80 (20-100)	83 (30-100)	90 (65-100)			
At least one GoCC during the pandemic||							0.26†		
Yes	13 (4)	4 (7)	3 (3)	3 (3)	3 (5)	0 (0)			
No	68 (21)	19 (32)	21 (24)	15 (15)	11 (19)	2 (13)			
*Two subjects with missing Moral Distress answers were excluded for all analyses; †Chi-Square p-value; ‡Kruskal-Wallis p-value; §Reported “Other” specialties include anticoagulation services, allergy and immunology, addiction medicine, cardiology, dermatology, endocrinology, employee health services, hematology/oncology, infectious disease, nephrology, neurology/traumatic brain injury, occupational health services, psychiatry/mental health, pain medicine, rheumatology, radiologic services, surgery, wound care, and unspecified; ||This item had 242 missing values.	
