# Supplementary material for: Addressing clinician moral distress: Implications from a mixed methods evaluation during Covid-19
Source: PLoS One. 2023 Sep 15;18(9):e0291542. doi: 10.1371/journal.pone.0291542 (PMC10503769; doi:10.1371/journal.pone.0291542)
Supplement: S5 Table — (DOCX) [file pone.0291542.s007.docx]

**S5 Table. Attitudes and Behaviors Related to Goals of Care Conversations**

|  |  |  |  |  |
| --- | --- | --- | --- | --- |
|  | Full Sample (N=321*)  n (%) | None/Mild/  Uncomfortable (N=247)  n (%) | Intense/  Severe (N=74)  n (%) | P-value |
| **How appropriate is it to provide an LST ^†^ recommendation?** |  |  |  | 0.18 |
| Very /Somewhat Appropriate | 268 (83) | 210 (85) | 58 (78) |  |
| Very/Somewhat Inappropriate | 53 (17) | 37 (15) | 16 (22) |  |
| **How comfortable are you with providing an LST recommendation?** |  |  |  | 0.61 |
| Very/Somewhat Comfortable | 233 (73) | 181 (73) | 52 (70) |  |
| Very/Somewhat Uncomfortable | 88 (27) | 66 (27) | 22 (30) |  |
| **How often do you ask COVID patients if they want your recommendations about LST decisions?** |  |  |  | 0.28 |
| Never | 57(24) | 46 (26) | 11 (18) |  |
| Rarely | 39(16) | 31(18) | 8 (13) |  |
| Sometimes | 56 (24) | 36 (20) | 20 (32) |  |
| Often | 48 (20) | 34 (19) | 14 (23) |  |
| Always | 38 (16) | 29 (16) | 9 (15) |  |
| **Providing a patient with a specific recommendation about LST is a healthcare provider’s duty** |  |  |  | 0.75 |
| Agree Strongly/Somewhat | 265 (83) | 203 (82) | 62 (84) |  |
| Disagree Strongly/Somewhat | 56 (17) | 44 (18) | 12 (16) |  |
| **Providing a patient with a specific recommendation about LST is appropriate only if the patient wants the recommendation** |  |  |  | 0.63 |
| Agree Strongly/Somewhat | 207 (64) | 161 (65) | 46 (62) |  |
| Disagree Strongly/Somewhat | 114 (36) | 86 (35) | 28 (38) |  |
| **Providing a patient with a specific recommendation about LST makes it easier for the patient to make LST decisions** |  |  |  | 0.31 |
| Agree Strongly/Somewhat | 280 (87) | 218 (88) | 62 (84) |  |
| Disagree Strongly/Somewhat | 41 (13) | 29 (12) | 19 (12) |  |
| **Providing a patient with a specific recommendation about LST further burdens the patient** |  |  |  | <0.001 |
| Agree Strongly/Somewhat | 61 (19) | 36 (15) | 25 (34) |  |
| Disagree Strongly/Somewhat | 260 (81) | 211 (85) | 49 (66) |  |
| **Providing a patient with a specific recommendation about LST unduly influences the patient** |  |  |  | <0.01 |
| Agree Strongly/Somewhat | 76 (24) | 49 (20) | 27 (36) |  |
| Disagree Strongly/Somewhat | 245 (76) | 198 (80) | 47 (64) |  |
| **Providing a patient with a specific recommendation about LST places too great a burden on the provider** |  |  |  | 0.01 |
| Agree Strongly/Somewhat | 67 (21) | 44 (18) | 23 (31) |  |
| Disagree Strongly/Somewhat | 254 (79) | 203 (82) | 51 (69) |  |
|  |  |  |  |  |
| **How ethically appropriate would it have been for a provider to discourage interventions by using vivid imagery (e.g., “CPR would break your ribs…”)?** |  |  |  | 0.94 |
| Definitely/Probably Appropriate | 127 (40) | 98 (40) | 29 (39) |  |
| Definitely Not/Probably Not Appropriate | 194 (60) | 149 (60) | 45 (61) |  |
| **How ethically appropriate would it have been to recommend a time-limited trial of therapy?** |  |  |  | 0.47 |
| Definitely/Probably Appropriate | 277 (86) | 215 (87) | 62 (84) |  |
| Definitely Not/Probably Not Appropriate | 44 (14) | 32 (13) | 12 (16) |  |
| **How ethically appropriate would it have been to focus the discussion on the medical facts that support the choice the provider thinks is best for the patient?** |  |  |  | 0.99 |
| Definitely/Probably Appropriate | 230 (72) | 177 (72) | 53 (72) |  |
| Definitely Not/Probably Not Appropriate | 91 (28) | 70 (28) | 21 (28) |  |
| **How ethically appropriate would it have been to divide the decision about limiting life support into smaller decisions about individual interventions?** |  |  |  | 0.88 |
| Definitely/Probably Appropriate | 275 (86) | 212 (86) | 63 (85) |  |
| Definitely Not/Probably Not Appropriate | 46 (14) | 35 (14) | 11 (15) |  |
| **How ethically appropriate would it have been to discuss the patient’s small chance of recovery?** |  |  |  | 0.41 |
| Definitely/Probably Appropriate | 286 (89) | 222 (90) | 64 (86) |  |
| Definitely Not/Probably Not Appropriate | 35 (11) | 25 (10) | 10 (14) |  |
| **How ethically appropriate would it have been to explain what the provider would do if the patient were the provider’s family member?** |  |  |  | 0.46 |
| Definitely/Probably Appropriate | 157 (49) | 118 (48) | 39 (53) |  |
| Definitely Not/Probably Not Appropriate | 164 (51) | 129 (52) | 35 (47) |  |
| **How ethically appropriate would it have been to emphasize those patient values that support the choice the provider thinks is best for the patient?** |  |  |  | 0.16 |
| Definitely/Probably Appropriate | 225 (70) | 178 (72) | 47 (64) |  |
| Definitely Not/Probably Not Appropriate | 96 (30) | 69 (28) | 27 (36) |  |
| **How ethically appropriate would it have been to discuss the provider’s prognostic uncertainty with the patient or family?** |  |  |  | 0.91 |
| Definitely/Probably Appropriate | 296 (92) | 228 (92) | 68 (92) |  |
| Definitely Not/Probably Not Appropriate | 25 (8) | 19 (8) | 6 (8) |  |
|  |  |  |  |  |
| **How ethically appropriate would it have been to make an independent treatment decision and inform the patient of that decision?** |  |  |  | 0.02 |
| Definitely/Probably Appropriate | 43 (13) | 27 (11) | 16 (5) |  |
| Definitely Not/Probably Not Appropriate | 278 (87) | 220 (89) | 58 (78) |  |
| **In general, how appropriate or inappropriate is it for providers to limit a COVID patient’s decision regarding LST because of the risk it poses on providers (e.g., inadequate personal protective equipment))?** |  |  |  | 0.04 |
| Definitely/Probably Appropriate | 68 (21) | 46 (19) | 22 (30) |  |
| Definitely Not/Probably Not Appropriate | 253 (79) | 201 (81) | 52 (70) |  |
| **In general, how appropriate or inappropriate is it for providers to limit a COVID patient’s decision regarding LST because of limited resources for other patients (e.g., ventilators, beds)?** |  |  |  | 0.13 |
| Definitely/Probably Appropriate | 132 (41) | 96 (39) | 36 (49) |  |
| Definitely Not/Probably Not Appropriate | 189 (59) | 151 (61) | 38 (51) |  |
| **How comfortable or uncomfortable are you with prognosticating about whether a patient without COVID will have an outcome consistent with their goals and values after respiratory failure?** |  |  |  | 0.33 |
| Very/Somewhat Comfortable | 224 (70) | 169 (68) | 55 (74) |  |
| Very/Somewhat Uncomfortable | 97 (30) | 78 (32) | 19 (26) |  |
| **How comfortable or uncomfortable are you with prognosticating about whether a patient with suspected of confirmed COVID will have an outcome consistent with their goals and values after respiratory failure?** |  |  |  | 0.63 |
| Very/Somewhat Comfortable | 164 (51) | 128 (52) | 36 (49) |  |
| Very/Somewhat Uncomfortable | 157 (49) | 119 (48) | 38 (51) |  |
| **Did you have at least one discussion with a patient about LST decisions during peak COVID?^‡^** |  |  |  | 0.71 |
| Yes | 13 (16) | 10 (15) | 3 (19) |  |
| No | 68 (84) | 55 (85) | 13 (81) |  |
| **After you have a goals of care conversation with a patient, how often do you feel confident that you provided the patient with adequate information to make a fully informed decision?** |  |  |  | 0.66 |
| Always | 61 (24) | 49 (26) | 12 (20) |  |
| Often | 160 (64) | 120 (63) | 40 (67) |  |
| Rarely/Never | 30 (12) | 22 (12) | 8(13) |  |
|  |  |  |  |  |
| **In general, how would you compare the quality of your goals of care conversations during peak COVID to those during pre-COVID?** |  |  |  | 0.14 |
| Much/Somewhat Better | 42 (17) | 29 (16) | 13 (22) |  |
| About the Same | 139 (58) | 111 (61) | 28 (47) |  |
| Much/Somewhat Worse | 60 (25) | 41 (23) | 19 (32) |  |
| **How do you think each of these situations affected the quality of goals of care conversations…**  **a. Restrictions on family/support system**  **presence during the conversation?** |  |  |  | 0.33 |
| Much/Somewhat Better | 12 (5) | 7 (4) | 5 (9) |  |
| About the Same | 21(10) | 17 (10) | 4 (7) |  |
| Much/Somewhat Worse | 188 (85) | 140 (85) | 48 (84) |  |
| **b. Communicating over the telephone?** |  |  |  | 0.77 |
| Much/Somewhat Better | 19 (8) | 13 (8) | 6 (11) |  |
| About the Same | 47 (20) | 35 (20) | 12 (21) |  |
| Much/Somewhat Worse | 162 (71) | 123 (72) | 39 (68) |  |
| **c. Communicating over video?** |  |  |  | 0.06 |
| Much/Somewhat Better | 21 (10) | 14 (9) | 7 (13) |  |
| About the Same | 61 (30) | 52 (34) | 9 (17) |  |
| Much/Somewhat Worse | 124 (60) | 87 (57) | 37 (70) |  |
| **How concerned were you about resource availability at your facility (e.g., personal protective equipment, beds, ventilators, staff)?** |  |  |  | <0.0001 |
| Extremely/Very/Moderately | 74 (23) | 46 (62) | 46 (62) |  |
| Slightly/Not At All | 247 (77) | 87 (35) | 28 (38) |  |

*Two subjects with missing Moral Distress answers were excluded for all analyses; ^†^ LST=Life Sustaining Treatment ; ^‡^ This item had 75 missing values.
